# Estimating conditional survival benefit for the allocation of scarce resources

**DOI:** 10.1177/09622802261420699

**Published:** 2026-02-17

**Authors:** Ilaria Prosepe, Nan van Geloven, Hans de Ferrante, Andries E Braat, Hein Putter

**Affiliations:** 1 Department of Biomedical Data Sciences, 4501 Leiden University Medical Center, Leiden, Zuid-Holland, the Netherlands; 2 Department of Mathematics and Computer Science, 3169 Eindhoven University of Technology, Eindhoven, Noord-Brabant, the Netherlands; 3 Eurotransplant International Foundation, Leiden, The Netherlands; 4 Department of Surgery, 4501 Leiden University Medical Center, Leiden, Zuid-Holland, the Netherlands

**Keywords:** Cross-sections, marginal structural models, inverse probability of treatment weighting, survival analysis, causal inference

## Abstract

Whenever treatment is scarce, the question of how to allocate resources arises. One option is to allocate based on conditional survival benefit, defined as the contrast between an individual’s expected survival with and without treatment. Estimating conditional survival benefit from observational data may present the following three challenges: (i) time-dependent confounding, arising when treatments are assigned based on longitudinal health markers which are in turn affected by past assignments; (ii) multiple time scales, namely time since becoming eligible for treatment, relevant for comparability of patients, and calendar time, representing when treatment decisions are made; and (iii) multiple versions of treatment, leading to multiple counterfactual outcomes with treatment. Building on previous work where cross-sections and inverse probability of treatment weighting were combined to estimate survival benefit on the treated population, we propose a strategy to dynamically estimate conditional survival benefit for all patients awaiting scarce treatment at any time treatment becomes available, accounting for multiple versions of treatment. After delineating identifiability assumptions, we show in a simulation study that our proposed method improves the estimation of conditional survival benefit compared to simpler methods. The proposed method is then applied to liver transplant data from the Eurotransplant region.

## 1. Introduction

Whenever treatment resources are scarce, difficult choices have to be made on which patients get to receive treatment. The allocation of scarce intensive care beds during the COVID-19 pandemic offers a good example. A second example, which motivates the current work, is the allocation of livers from deceased organ donors. The scarcity of donor livers results in a waiting list of patients who are considered eligible for transplantation and in a need of a prioritization rule on who is offered a donor liver when one becomes available. Since 2006, the Model for End-stage Liver Disease (MELD) score has been used in the Eurotransplant region to prioritize patients. The score is calculated based on values of serum creatinine, bilirubin and international normalized ratio (INR) of the prothrombin time, which are routinely collected together with other markers to monitor the clinical status of the patients. The MELD score is a strong indicator of disease severity and waiting list mortality and helps keeping track, throughout time, of those who are at highest risk of pre-transplant death. However, this score does not take post-transplant mortality into account. Lately there has been increasing interest in basing organ allocation on the individual expected survival benefit, that is, the predicted number of life-years gained due to transplant for an individual,^1^ with the United Kingdom recently implementing his approach to prioritize patients awaiting transplantation.^2^ More precisely, survival benefit can be quantified as the difference between two conditional counterfactual survival outcomes: the restricted mean survival time when receiving the transplant minus the restricted mean survival time when not receiving it. Estimating the conditional expected survival benefit can help clinicians make informed decisions on prioritization of patients.

There are three main issues that arise while estimating survival benefit. The first issue is confounding. In the motivating example, patients begin their follow-up untreated and eventually receive treatment (transplantation) or die untreated. In this context, the survival benefit of interest becomes the contrast of: (i) patient’s survival if they remain untreated, conditional on having survived, untreated, up until the moment of potential treatment and conditional on the patient’s health up to that moment (never-treated survival); (ii) patient’s survival if they do receive the available treatment (with its specific characteristics), conditional on having survived, untreated, up until the moment of potential treatment and conditional on patient-specific covariates evaluated at time of treatment (post-treatment survival). To avoid confounding, these two components should ideally be estimated on data collected via conditionally randomized trials, that is, trials where randomization is guaranteed within subgroups of the population.^3^ However, randomization is uncommon in the field of transplantation and we therefore need to rely on the observational data at hand, where the relation between transplantation and survival is heavily confounded by MELD score. In particular, MELD changes through time and consequently influences both transplantation and survival chances; in return, MELD is influenced by the treatment choice, as the patient’s health usually keeps deteriorating while on the waiting list. In order to avoid bias due to this time-dependent confounding, adjustment techniques (such as inverse probability weighting) are needed.^4^

The second issue arises from the fact that treatment is a scarce resource which only becomes available irregularly at certain calendar times. Decisions on the prioritization of patients have to be made in calendar time, whenever a new treatment resource becomes available. Hence, in order to prioritize based on survival benefit, we need a model that can correctly estimate survival benefit based on the most recent information we have on the patients’ health at any given calendar date. In previous work,^5^ the use of a partly conditional method was proposed for this setting where clinical decisions need to be made in calendar time. This method is closely related to other approaches such as the sequential trial approach^6,7^ and the sequential stratification approach,^8–10^ which all involve the creation of artificial time origins to analyze the data. In Gong and Schaubel,^5^ a version of inverse probability of censoring weighting tailored to the sequential stratification methodology is proposed to estimate never-treated survival from moment of potential treatment onward. In a later work by the same authors,^11^ these methods are further explored to estimate average survival benefit on the treated population, by contrasting the average counterfactual never-treated survival to the average factual post-transplant survival on the treated population.

The third issue concerns the presence of multiple versions of treatment in the estimation of the counterfactual post-transplant survival. Estimating counterfactual survival risks in presence of confounding requires causal inference methods, which rely on several assumptions.^3^ A key assumption is the no-multiple-versions-of-treatment assumption,^3,12^ which assumes that a potential outcome under a given treatment is a single well-defined value. In practice, however, this assumption may be violated. For instance, in liver transplantation treatments (livers) can differ in quality and potentially lead to different outcomes. Accounting for multiple versions of treatment represents a challenge which is not present in the work by Gong and Schaubel.^5,11^ Their approach estimated survival benefit among the treated and therefore did not involve modeling counterfactual post-transplant survival—only the factual survival observed in treated patients. The only counterfactual quantity required for their analysis was the never-treated survival, used to estimate what survival would have been had treated patients not received a transplant. Unlike post-transplant survival, never-treated survival has only one version, as there are no variations in “non-treatment,” and thus does not require addressing multiple treatment versions.

In this work, we build upon the partly conditional method with adjustment for time-dependent confounding proposed by Gong and Schaubel^5,11^ to estimate conditional survival benefit in a way that applies to *all* patients awaiting treatment, rather than only those who have been treated, thereby providing a quantity that can inform clinical decision-making. Following the framework of Gong and Schaubel,^5,11^ we combine marginal structural models (MSMs) with multiple baselines, ensuring that the model can be used to make predictions not only at one fixed moment but repeatedly in calendar time—aligning with the type of predictions needed to support clinical decisions among patients on a waiting list. We extend the approach to (i) ensure applicability to the entire waiting list population, and (ii) account for multiple versions of treatment. These extensions introduce additional complexities. In this article, we clearly specify the assumptions required for identification of conditional survival benefit and propose an estimation procedure. Specifically, we introduce a reweighting strategy that enables the construction of an MSM capable of estimating both never-treated and post-treatment survival for the entire waiting list population, while explicitly accounting—under clearly stated assumptions—for multiple versions of treatment.

In Section 2, we introduce the notation, establish identifiability results, and describe the proposed estimation method. In Section 3, the practical validity of the proposed method is evaluated through a simulation study. In Section 4, we apply the proposed method to liver transplant data. In Section 5, we discuss our findings.

## 2. Methods

### 2.1. Notation

We start by establishing the set-up and the notation, which is similar to the one proposed by Gong and Schaubel.^5^ Let t be follow-up time, which starts at t=0 when a patient is first considered eligible for treatment. We let D be time from first eligibility to death and C be censoring time. We define the observed survival time as X=min(D,C) and the status indicator for death as Δ=I(X=D) . For patients that receive treatment, we denote the time of treatment by T . We define the time-dependent treatment indicator for any treatment as A(t)=I(t≥T) and the time-dependent indicator for treatment type ℓ∈L as $A_{\ell }(t)$ which takes value 1 if A(t)=1 and treatment version received is ℓ and 0 otherwise. We denote the treatment history by $\bar{A}(t)=\{A(t^{\prime });t^{\prime }\in [0,t]\}$ and the history for treatment type ℓ ${\bar{A}}_{\ell }(t)=\{A_{\ell }(t^{\prime });t^{\prime }\in [0,t]\}$ . We write $\bar{A}(t)\equiv 0$ ( ${\bar{A}}_{\ell }(t)\equiv 0$ ) to indicate $A(t^{\prime })=0$ ( $A_{\ell }(t^{\prime })=0$ ) for $t^{\prime }\in [0,t]$ . Finally, we let Z(t) be the time-dependent patient covariates and $\bar{Z}(t)=\{Z(t^{\prime });t^{\prime }\in [0,t]\}$ be the covariate history. While $\bar{A}(t)\equiv 0$ , the patient’s survival at time t is influenced by $\bar{Z}(t)$ ; when A(t)=1 , we assume that the patient’s survival at time t is influenced by $\bar{Z}(T)$ and by the treatment type ℓ . To accommodate the fact that patients may at some times be unfit or unavailable for treatment, we also introduce E(t) as the time-dependent eligibility indicator, assuming the value 1 if the patient is eligible for treatment just before time t and 0 otherwise, and $\bar{E}(t)=\{E(t^{\prime });t^{\prime }\in [0,t]\}$ as the eligibility history. Note that if E(t) takes value one, this implies that the patient is still alive and untreated just before time t as the patient is only eligible while still alive, awaiting for treatment (which can only be initiated once) and uncensored. This means that E(t)=0 for t>min{X,T} . We assume throughout that the patient’s history $\bar{Z}(t)$ includes the eligibility history $\bar{E}(t)$ as well as the follow-up time t .

Now let us consider K equally-spaced calendar cross-section dates $\{{\text{CS}}_{k}{\}}_{k=1,\ldots ,K}$ , representing the time when a treatment becomes available and a decision has to be made on treatment, as described by Gong and Schaubel.^5^ We are interested in modeling survival from these dates onward, resetting the clock at each of these calendar dates. We fix some notation to accommodate this time reset. For each cross-section ${\text{CS}}_{k}$ , we let $S_{k}$ be the time from first eligibility to ${\text{CS}}_{k}$ . Note that, if we only consider those patients who are eligible for treatment at ${\text{CS}}_{k}$ (i.e. $E(S_{k})=1$ ), $S_{k}$ is non-negative. We can therefore define $u:=t-S_{k}$ , which represents time since cross-section ${\text{CS}}_{k}$ . This yields the following times since cross-sections: $T_{k}=T-S_{k}$ , $D_{k}=D-S_{k}$ , and $X_{k}=X-S_{k}$ . These times are non-negative as $S_{k}\leq X_{k}$ . We define the status indicator $\Delta_{k}=I(X_{k}=D_{k})$ , the treatment indicator $A_{k}(u)=I(u\geq T_{k})$ , with realization $a_{k}(u)$ , and the treatment-specific indicator $A_{k,\ell }(u)$ , with realization $a_{k,\ell }(u)$ , which takes value 1 if $A_{k}(u)=1$ and the treatment type received is ℓ and 0 otherwise. Treatment history for any treatment (for treatment type ℓ ) is ${\bar{A}}_{k}(u)=\{A_{k}(u^{\prime }):u^{\prime }\in [0,u]\}$ ( ${\bar{A}}_{k,\ell }(u)=\{A_{k,\ell }(u^{\prime }):u^{\prime }\in [0,u]\}$ ). We write ${\bar{A}}_{k}(u)\equiv 0$ ( ${\bar{A}}_{k,\ell }(u)\equiv 0$ ) to indicate $A_{k}(u^{\prime })=0$ ( $A_{k,\ell }(u^{\prime })=0$ ) for $u^{\prime }\in [0,u]$ . Finally, we fix the following notation for the covariates: $E_{k}=E(S_{k})$ , $Z_{k}=Z(S_{k})$ , ${\bar{Z}}_{k}=\bar{Z}(S_{k})$ , and ${\bar{Z}}_{k}(u)=\bar{Z}(S_{k}+u)$ .

In this work, we aim to contrast two treatment strategies for patients who are still eligible (meaning alive, untreated and available to initiate treatment) at time $S_{k}$ : (i) never receiving treatment and (ii) receiving treatment type ℓ at time $S_{k}$ . We let ${\underline{a}}_{k,\ell }(u)=\{a_{k,\ell }(u^{\prime }),u^{\prime }\geq u\}$ describe treatment strategies from cross-section k . We write ${\underline{a}}_{k,\ell }(0)\equiv 1$ if $a_{k,\ell }(u)=1\text{ for all }u^{\prime }\geq u$ and ${\underline{a}}_{k}(0)\equiv 0$ if $a_{k,\ell }(u)=0\text{ for all }u^{\prime }\geq u$ and for every ℓ . Under this notation, the two treatment strategies of interest at a certain time treatment type ℓ becomes available are ${\underline{a}}_{k}(0)\equiv 0$ and ${\underline{a}}_{k,\ell }(0)\equiv 1$ , respectively. We reflect on further possible treatment strategies in the discussion.

We define $D_{k}^{{\underline{a}}_{k}(0)\equiv 0}$ , the potential residual time to death from time $S_{k}$ if treatment is never initiated, and $D_{k}^{{\underline{a}}_{k,\ell }(0)\equiv 1}$ , the potential post-treatment residual time to death from time $S_{k}$ if treatment type ℓ is initiated at time $S_{k}$ . For simplicity, we will refer to these two outcomes as $D_{k}^{0}$ and $D_{k}^{1,\ell }$ , respectively. We remark that $D_{k}^{0}+S_{k}$ is the same for all values of k and we refer to this quantity as $D^{0}$ , which is time to death under no treatment since first eligibility. We define the two potential residual survival distributions (i) and (ii) as follows:

(1) $$\begin{array}{rl} S^{0}(u,S_{k}\;|\;{\bar{Z}}_{k}) & :=P\{D_{k}^{0}>u\;|\;{\bar{Z}}_{k},E_{k}=1\}\\ S^{1,\ell }(u,S_{k}\;|\;{\bar{Z}}_{k}) & :=P\{D_{k}^{1,\ell }>u\;|\;{\bar{Z}}_{k},E_{k}=1\} \end{array}$$

For these two quantities, the mean survival times, restricted to some pre-specified fixed time horizon L , can be defined as follows:

(2) $$\begin{array}{rl} {\text{RMST}}^{0}(S_{k}\;|\;{\bar{Z}}_{k}) & :=\int_{0}^{L}S^{0}(u,S_{k}\;|\;{\bar{Z}}_{k})du\\ {\text{RMST}}^{1,\ell }(S_{k}\;|\;{\bar{Z}}_{k}) & :=\int_{0}^{L}S^{1,\ell }(u,S_{k}\;|\;{\bar{Z}}_{k})du \end{array}$$

**Figure 1.**
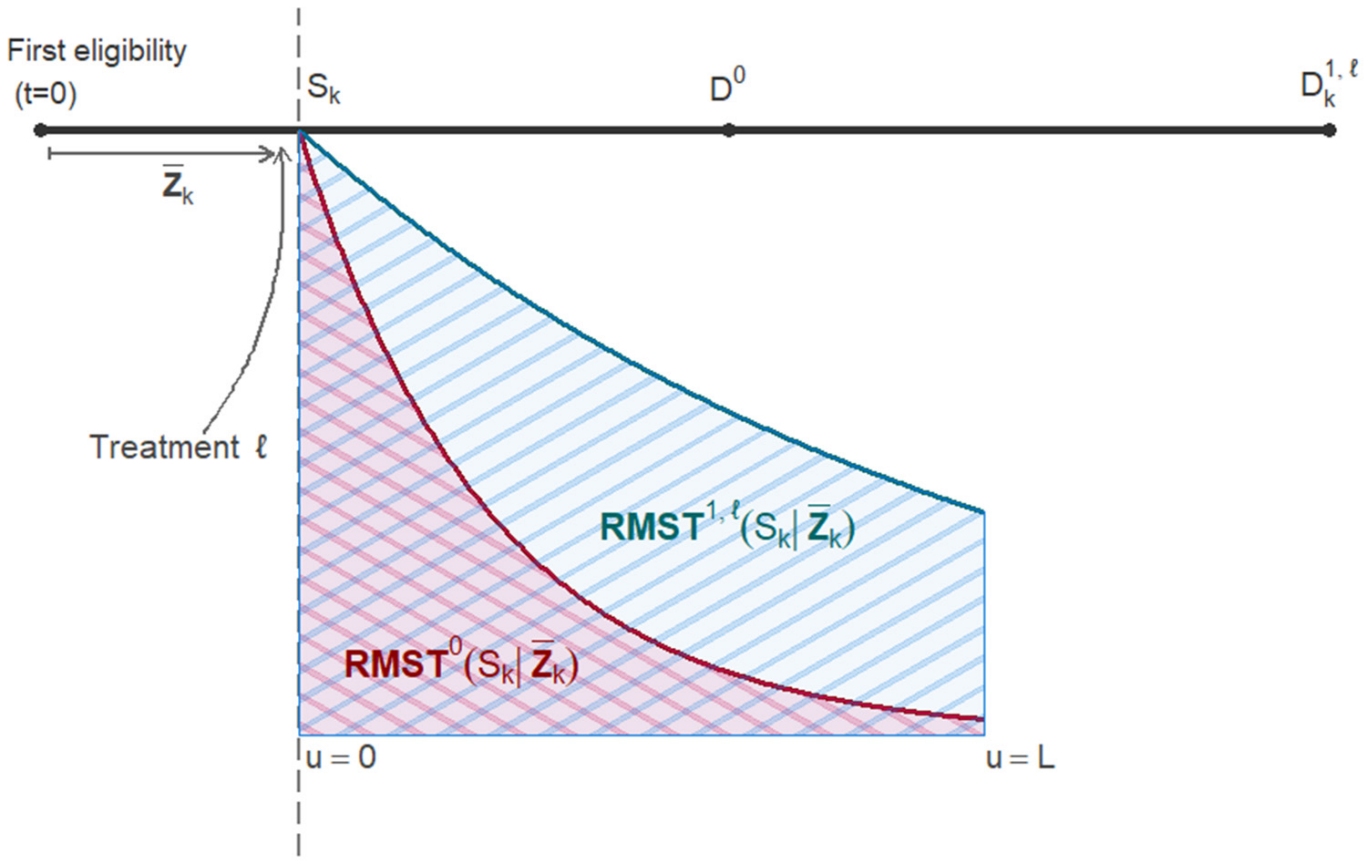
Graphical depiction of the notation used in the article. We consider one patient awaiting treatment. Time t represents time since first eligibility, $S_{k}$ is the fixed time point corresponding to cross-section ${\text{CS}}_{k}$ , ${\bar{Z}}_{k}$ is the patient’s covariate history, $D^{0}$ is the potential time to death if treatment is never assigned and $D_{k}^{1,\ell }$ is the potential time to death if treatment type ℓ is initiated at time $S_{k}$ . The red curve represents the residual survival distribution from $S_{k}$ onward if treatment is never assigned, and the blue curve represents the residual survival distribution from $S_{k}$ onward if treatment type ℓ is administered at time $S_{k}$ . The areas under the two survival curves yield respectively the restricted mean survival time (RMST) from u=0 to the prediction horizon u=L with and without treatment, where $u:=t-S_{k}$ .

Figure 1 provides a graphical depiction of the two restricted mean survival times ${\text{RMST}}^{0}(S_{k}\;|\;{\bar{Z}}_{k})$ and ${\text{RMST}}^{1,\ell }(S_{k}\;|\;{\bar{Z}}_{k})$ . We define the conditional survival benefit of treatment type ℓ as the difference between the restricted mean survival times (RMSTs) as follows:

(3) $$d^{\ell }(S_{k}\;|\;{\bar{Z}}_{k}):={\text{RMST}}^{1,\ell }(S_{k}\;|\;{\bar{Z}}_{k})-{\text{RMST}}^{0}(S_{k}\;|\;{\bar{Z}}_{k})$$

### 2.2. Identifiability

Ideally, the quantities in equations (1) to (3) would be estimated from a sequentially randomized trial. In such a trial, at each cross-section k , eligible patients would be randomly assigned to either initiate or not initiate the available treatment type ℓ , with randomization stratified by patient characteristics. However, if such a trial is not ethical or feasible, this necessitates analyses of observational data. In this section, we outline three identifiability conditions that allow us to give causal interpretation to estimates obtained from observational data such as described above.

The three identifiability conditions are:

- consistency:
  – the counterfactual outcome $D_{k}^{0}$ is equal to the observed outcome $D_{k}$ for those patients who die untreated; we write $D_{k}^{0}=(D_{k}\;|\;\bar{A}(D_{k})\equiv 0)$ ;
  – the counterfactual outcome $D_{k}^{1,\ell }$ is equal to the observed outcome $D_{k}$ for those patients who are treated at cross-section k with treatment type ℓ ; we write $D_{k}^{1,\ell }=(D_{k}\;|\;A_{k,\ell }(0)=1)$ ;
- positivity:
  – the probability of being treated at time t given $\bar{Z}(t)$ , $E_{k}=1$ , $A(t^{-})\equiv 0$ and D≥t is <1 for all t ;
  – the probability of being treated at ${\text{CS}}_{k}$ with treatment type ℓ given $E_{k}=1$ and ${\bar{Z}}_{k}$ is >0;
- conditional exchangeability:
  – for every u≥0 : $D_{k}^{0}⊥⊥A_{k}(u)\;|\;{\bar{Z}}_{k}(u),A_{k}(u^{-})\equiv 0,D_{k}\geq u,E_{k}=1$ ;
  –
$D_{k}^{1,\ell }⊥⊥A_{k,\ell }(0)\;|\;{\bar{Z}}_{k},E_{k}=1$ .

Under these conditions, the survival distributions in equation (1) become identifiable. A proof of this main result can be found in Section A.1 of the Supplemental Material. The proof shows that these survival distributions can be expressed in terms of hazard functions and weights. The hazard functions are defined as follows:

(4) $$\begin{array}{rl} \lambda_{0,k}(u\;|\;{\bar{Z}}_{k}) & =\lim_{\delta \to 0}\frac{1}{\delta }P[u\leq D_{k}<u+\delta \;|\;{\bar{Z}}_{k},E_{k}=1,D_{k}\geq u,{\bar{A}}_{k}(u^{-})\equiv 0]\\ \lambda_{1,k,\ell }(u\;|\;{\bar{Z}}_{k}) & =\lim_{\delta \to 0}\frac{1}{\delta }P[u\leq D_{k}<u+\delta \;|\;{\bar{Z}}_{k},E_{k}=1,D_{k}\geq u,A_{k,\ell }(0)=1] \end{array}$$

and the weights correspond to the inverse probability of having followed the treatment strategies ${\underline{a}}_{k}(0)\equiv 0$ and ${\underline{a}}_{k,\ell }(0)\equiv 1$ , respectively, up to the time horizon L . To put it in simpler words, the survival distributions at (1) can be estimated on a pseudo-population where subjects are artificially censored as soon as they deviate from the respective treatment strategies and reweighted by the inverse probability of keep on adhering to those strategies over time. If it can be assumed that the censoring mechanism that was already present in the data is independent of $D_{k}$ conditional on ${\bar{Z}}_{k}$ , the hazards at equation (4) can be estimated from data without additional reweighting. Otherwise, under some conditions, it is still possible to estimate from observational data, but additional reweighting is required. See Section A.2 of the Supplemental Material for the exact formulation of the conditions and the weights.

### 2.3. Estimation

We choose the set of covariates $Z_{k}$ in such a way that they are indicative of the covariate history and we assume a bijective mapping between treatment quality L and a set of treatment covariates $Z^{*}$ . We denote by $z_{\ell }^{*}$ the values of the covariates corresponding to treatment level ℓ . We assume $\lambda_{1,k,\ell }(u\;|\;{\bar{Z}}_{k})=\lambda_{1,k}(u\;|\;Z_{k},Z^{*}=z_{\ell }^{*})$ can be estimated, in the reweighted pseudo-population, by pooling together all patients with $A_{k}(0)=1$ and using $Z_{k}$ and $Z^{*}$ (and possibly their interactions) as covariates. Furthermore we assume that death times can be modeled via Cox regression. Then (4) becomes

(5) $$\begin{array}{rl} \lambda_{0,k}(u\;|\;{\bar{Z}}_{k}) & =E_{k}\lambda_{0,0,k}(u)\exp\;\left(f_{0}(Z_{k};\;\beta_{0})\right)\\ \lambda_{1,k,\ell }(u\;|\;{\bar{Z}}_{k}) & =E_{k}\lambda_{0,1,k}(u)\exp\;\left(f_{1}(Z_{k};\;Z^{*}=z_{\ell }^{*};\;\beta_{1})\right) \end{array}$$

where $\beta_{0}$ is the (possibly time-dependent) vector of coefficients for mortality without treatment, $\beta_{1}$ is the (possibly time-dependent) vector of coefficients for mortality with treatment, and $\lambda_{0,0,k}(u)$ and $\lambda_{0,1,k}(u)$ are the baseline hazards. If we assume proportionality and linearity, these equations reduce to $\lambda_{0,k}(u\;|\;{\bar{Z}}_{k})=E_{k}\lambda_{0,0,k}(u)\exp\;(\beta_{0}Z_{k})$ and $\lambda_{1,k}(u\;|\;{\bar{Z}}_{k},Z^{*})=E_{k}\lambda_{0,1,k}(u)\exp\;(\beta_{1}(Z_{k};Z^{*}))$ .

To construct the weights for creating the pseudo-population, we extend the stabilized weights suggested by Gong and Schaubel^5^ to also include patients that do receive treatment. This requires two models for the denominator. The first one estimates the probability of remaining untreated based on a patient’s history, used to reweight untreated patients. The second one estimates the probability of receiving treatment type ℓ at ${\text{CS}}_{k}$ given the patient’s history, $P\{A_{k,\ell }(0)=1\;|\;{\bar{Z}}_{k},E_{k}=1\}$ , used to reweight treated patients. Because the type of treatment becoming available at ${\text{CS}}_{k}$ is independent from ${\bar{Z}}_{k}$ , $P\{A_{k,\ell }(0)=1\;|\;{\bar{Z}}_{k},E_{k}=1\}$ is proportional, in each cross-section, to $P\{A_{k}(0)=1\;|\;{\bar{Z}}_{k},Z^{*}=z_{\ell }^{*},E_{k}=1\}$ . Since at ${\text{CS}}_{k}$ all eligible patients are reweighed by the probability of receiving that specific treatment type, we can reweight by $P\{A_{k}(0)=1\;|\;{\bar{Z}}_{k},Z^{*}=z_{\ell }^{*},E_{k}=1\}$ , which can be estimated using $Z^{*}$ as covariate.

We model the first probability, that is, the probability of remaining untreated given patient’s history, using Cox regression. Weights are often estimated using a pooled logistic regression, which, in our context where treatment is only administered once and that those who are treated stay in the treated condition from then on, is asymptotically equivalent to a Cox regression with time-dependent covariates.^13^ We model the second probability, that is, the probability of receiving treatment at ${\text{CS}}_{k}$ , given the patient’s history and treatment characteristics, using complementary log-log regression. To stabilize the weights, we model the probability of remaining untreated given patient’s history until ${\text{CS}}_{k}$ using Cox regression for the untreated patients and the probability of receiving treatment at ${\text{CS}}_{k}$ for the treated patients. We obtain the following weights:

(6) $$W_{k}(u\;|\;{\bar{A}}_{k}(u),\bar{Z}(u+S_{k}),Z^{*})=\left\{ \begin{array}{ll} \frac{\exp\;(-\int_{0}^{u}\lambda_{0,\text{num},k}(u^{\prime }\;|\;Z_{k})du^{\prime })}{\exp\;(-\int_{S_{k}}^{S_{k}+u}\lambda_{0,\text{den}}(t^{\prime }\;|\;\bar{Z}(t^{\prime }))dt^{\prime })} & \text{ if }{\bar{A}}_{k}(u)\equiv 0\\ \frac{\lambda_{1,\text{num},k}}{1-\exp\;\{-\lambda_{1,\text{den},k}({\bar{Z}}_{k},Z^{*})\}} & \text{ if }{\bar{A}}_{k}(u)\equiv 1 \end{array}\right.$$

where

(7) $$\begin{array}{rl} \lambda_{0,\text{den}}(t\;|\;\bar{Z}(t)) & =E(t)\lambda_{0,\text{den}}(t)\exp\;\{f_{0,\text{den}}(Z(t);\beta_{0,\text{den}})\}\\ \lambda_{0,\text{num},k}(u\;|\;Z_{k}) & =E_{k}\lambda_{0,\text{num},k}(u)\exp\;\{f_{0,\text{num}}(Z_{k};\beta_{0,\text{num}})\}\\ \lambda_{1,\text{den},k}({\bar{Z}}_{k},Z^{*}) & =E_{k}\cdot \beta_{1,\text{den},k}\exp\;\{f_{1,\text{den}}(Z_{k},Z^{*},Z_{k}\cdot Z^{*};\beta_{1,\text{den}})\} \end{array}$$

and $\lambda_{1,\text{num},k}$ is the probability of receiving treatment at each cross-section ${\text{CS}}_{k}$ , which can be used to stabilize the weights. In these hazards, $\beta_{\text{den}}$ and $\beta_{\text{num}}$ are the (possibly time-dependent) vectors of coefficients and $\lambda_{0,\text{den}}(t\;|\;\bar{Z}(t))$ and $\lambda_{0,\text{num},k}(u\;|\;Z_{k})$ are the baseline hazards. A simple choice for $f_{0,\text{den}}(Z(t);\beta_{0,\text{den}})$ , $f_{0,\text{num}}(Z_{k};\beta_{0,\text{num}})$ , and $f_{1,\text{den}}(Z_{k},Z^{*},Z_{k}\cdot Z^{*};\beta_{1,\text{den}})$ would be respectively, assuming linearity and proportional hazards, $\beta_{0,\text{den}}Z(t)$ , $\beta_{0,\text{num}}Z_{k}$ , and $\beta_{1,\text{num}}\cdot (Z_{k},Z^{*},Z_{k}\cdot Z^{*})$ .

We note that the denominator of the weights presented in the first line of equation (6) represents the simplest function to obtain a pseudo-population on which $S^{0}(u,S_{k}\;|\;{\bar{Z}}_{k})$ can be estimated from the hazard function described at equation (4) (see Section A of the Supplemental Material). This formulation allows for different baseline hazards for each cross-section, but we assume that the covariates have the same effect in all cross-sections. However, any suitably defined function $g(Z_{k})$ may be included in the numerator of the weights to obtain the same estimates but with less variance.^3^ We choose this particular form of $g(Z_{k})$ based on the findings of the simulation study by Gong and Schaubel.^5^ The results of their study show that this choice leads to reduced variance in the estimates of $S^{0}(u,S_{k}\;|\;{\bar{Z}}_{k})$ .

For the weights presented in the second line of equation (6), we note that in a setting of scarce resources, it is likely that only a small number of patients are treated at each cross-section. This can lead to challenges in estimating $\beta_{1,\text{den},k}$ . To address this, one option is to pool cross-sections that are believed to have similar patient compositions, allowing for a shared intercept across those sections. Alternatively, the change in $\beta_{1,\text{den},k}$ across cross-sections could be modeled parametrically using splines, capturing potential trends over time.

## 3. Simulation

### 3.1. Simulation setup

In this section, we will follow the planning and reporting approach for simulation studies proposed by Morris et al.^14^

#### 3.1.1. Aim

The aim of the simulation is to evaluate if the proposed method can estimate accurately the quantities specified in equation (2), for all patients on the waiting list, at all cross-sections. The proposed method is compared to simpler modeling approaches.

#### 3.1.2. Data-generating mechanism

Consistently with the previous section, we use t for follow-up time, in years. Moreover, we define calendar time s≥0 as time since the beginning of the study. We generate data for an observational window that ranges from s=0 to s=10 years. Note that, other than generating observational data, we also need to generate the “truth,” which we will compare our estimated values to. With this goal in mind, we define the maximum time for which we need values of the time-dependent covariate $t_{\max}=10+L$ years, where L=3 years is the time horizon.

We start by generating all potential outcomes. For each patient, we generate a time of first eligibility $s^{\text{entry}}∼\text{Unif}(0,10)$ and longitudinal values of one time-dependent covariate, for $0\leq t\leq t_{\max}$ , as

(8) Z(t)=μ+ηt+ξ(t)

where μ∼N(0,1) , η∼N(0,0.5) , and ξ(t) is a mean zero Ornstein-Uhlenbeck (OU) process, similar to Putter and Houwelingen.^15^ This OU process is defined by $\xi (0)=\xi_{0}$ , and dξ(t)=−θξ(t)dt+σdW(t), where W(t) is a Wiener process and θ=0.1 and σ=0.0045 are, respectively, the degree of mean reversal and influence of the random fluctuations of the Wiener process. The value $\xi (0)=\xi_{0}$ is taken to be the $100^{\text{th}}$ burn-in of the OU process, starting from a baseline value of 0. After having generated the trajectory of Z(t) for $0\leq t\leq t_{\text{max}}$ , the potential time to death if a patient would remain untreated, $D^{0}$ , is drawn from a survival distribution with hazard $h^{0}(t\;|\;Z(t))=\lambda_{0}(t)\exp\;(\beta_{0}Z(t))$ , where $\lambda_{0}(t)=0.2t$ and $\beta_{0}=1.5$ represents the effect of Z(t) . Patient’s treatment chances also vary over time based on Z(t) and we draw T based on the hazard $h^{T}(t\;|\;Z(t))=\lambda_{T}(t)\exp\;(\beta_{T}Z(t)),$ where $\lambda_{T}(t)=0.25t$ and $\beta_{T}=2$ represents the effect of Z(t) on treatment chances. Differently from $D^{0}$ and T , we draw $D^{1}$ based on the subject’s condition at time of treatment and treatment covariate, with hazard $h^{1}(t-T|Z(T),Z^{\tau })=\lambda_{1}(t-T)\cdot \exp\;(f(Z(T))+\gamma_{1}Z^{*})$ for t>T , where $\lambda_{1}(t-T)=0.075(t-T)$ , $Z^{*}∼N(0,1)$ is the treatment-specific covariate that represents the quality of the treatment that becomes available at the calendar date which corresponds to time T , f(Z(T)) is the non-linear function which expresses the effect of Z(T) , and $\gamma_{1}=1$ represent the effect of $Z^{*}$ . We define f(x) as taking value 1.2 for x≤−0.6 , −2x for −0.6<x<0.6 , and − 1.2 for x≥0.6 . The choice to include Z(T) non-linearly is motivated by the wish to make the relation between Z(T) and survival different for patients who are more likely to receive treatment compared to those who are less likely to receive it. We remark that, for simplification, in this data-generating mechanism, $h^{T}(t\;|\;Z(t))$ does not depend on $Z^{*}$ , meaning that $Z^{*}$ is not a confounder for the relation between time-to-treatment and survival after treatment.

We then move on to generating the observational data that we use for estimation. For each patient, we generate $X=\min\{D^{0},T,10-s^{\text{entry}}\}$ . If X=T , the patient is treated, and the observed data consists of $s^{\text{entry}}$ , T , $D^{1}$ , $\bar{Z}(T)$ , and $Z^{*}$ ; if $X=D^{0}$ , the patient dies untreated, and the observed data consists of $s^{\text{entry}}$ , $D^{0}$ , and $\bar{Z}(D^{0})$ ; if $X=10-s^{\text{entry}}$ , the patient is censored before treatment, and the observed data consists of $s^{\text{entry}}$ , X , and $\bar{Z}(X)$ . We also generate the cross-sections $\{{\text{CS}}_{k}{\}}_{k=1,\ldots ,K}$ , as a set of evenly spaced (every 0.04 years) calendar dates, such that $0\leq {\text{CS}}_{k}<9$ . For each cross-section ${\text{CS}}_{k}$ , we generate one treatment specific covariate $Z_{k}^{*}∼N(0,1)$ , consistently with the $Z^{*}$ ’s generated previously. This means that those cross-sections which correspond to a calendar date for which a treatment-specific covariate already exists will take that value, while for the remaining cross-sections new $Z_{k}^{*}$ will be drawn. We also generate a finer set of cross-sections $\{{\text{CS}}_{k^{\prime }}{\}}_{k^{\prime }=1,\ldots ,K^{\prime }}$ (every 0.01 years), which will be employed uniquely for the estimation of the survival with treatment. Patients are included in a cross-section ${\text{CS}}_{k}$ (or ${\text{CS}}_{k^{\prime }})$ if the value of the time-dependent covariate is >− 1. The rationale behind this choice is that patients in better health at a cross-section are removed from the waiting list due to improvement, making them no longer eligible for treatment. This choice keeps the distribution of Z(t) among those on the waiting list approximately stable over time.

#### 3.1.3. Estimand

The aim of the simulation is to evaluate if the proposed method can correctly estimate (2), for all patients on the waiting list, at all cross-sections. Hence, in each replication, the goal is to estimate ${\text{RMST}}^{0}(S_{k}\;|\;{\bar{Z}}_{k})$ and ${\text{RMST}}^{1,\ell_{k}}(S_{k}\;|\;{\bar{Z}}_{k})={\text{RMST}}^{1}(S_{k}\;|\;{\bar{Z}}_{k},Z_{k}^{*}=z_{k,\ell }^{*})$ , where $S_{k}$ is the time between $s^{\text{entry}}$ and ${\text{CS}}_{k}$ and $\ell_{k}$ is the treatment type corresponding to realization $z_{k,\ell }^{*}$ of the covariate $Z_{k}^{*}$ , for each patient and for each k and compare the estimated values to the truth. The true values of the RMST s are computed as follows:

(9) $$\begin{array}{rl} {\text{RMST}}^{0}(S_{k}\;|\;Z(t)) & :=\int_{0}^{L}\exp\;(-\int_{S_{k}}^{u}h^{0}(s\;|\;Z(s))ds)du\\ {\text{RMST}}^{1}(S_{k}\;|\;Z_{k},Z_{k}^{*}) & :=\int_{0}^{L}\exp\;(-\int_{0}^{u}h_{S_{k}}^{1}(s\;|\;Z_{k},Z_{k}^{*})ds)du \end{array}$$

where $h_{S_{k}}^{1}(u\;|\;Z_{k},Z_{k}^{*})=\lambda_{1}(u)\exp\;(\beta_{1}Z_{k}+\gamma_{1}Z_{k}^{*})$ . Note that ${\text{RMST}}^{0}(S_{k}\;|\;Z(t))$ does not directly correspond with the target estimand ${\text{RMST}}^{0}(S_{k}\;|\;{\bar{Z}}_{k})$ , but is the true “individual” RMST from time $S_{k}$ for a subject with covariate history Z(t) . This quantity can be computed via numerical integration and is therefore more straightforward to obtain than the latter. The marginalization over all possible future trajectories of Z(t) after $S_{k}$ is performed implicitly through the choice of appropriate performance measures (see below). The true value of benefit is computed as follows:

(10) $$d(S_{k}\;|\;Z(t),Z_{k},Z_{k}^{*}):={\text{RMST}}^{1}(S_{k}\;|\;Z_{k},Z_{k}^{*})-{\text{RMST}}^{0}(S_{k}\;|\;Z(t))$$

#### 3.1.4. Methods

In our simulation study, we compare the performance of three methods which are here summarized briefly; for more detail see Section B in the Supplemental Material. The three methods are (i) the *naive* method, where ${\text{RMST}}^{0}$ is estimated via a Cox model with Z(0) as covariate, without any adjustment and ${\text{RMST}}^{1}$ is estimated via a Cox model with Z(T) as covariate, without any adjustment; (ii) the *cross-sections unweighted* method, which follows the same modeling approach as in the proposed method, but without the use of time-dependent weights; and (iii) the *cross-sections weighted* method, which is the proposed method. For the *cross-sections weighted* method and the *cross-sections unweighted* method, we use the set of cross-sections ${\text{CS}}_{k}$ for the estimation of survival without treatment and the set of cross-sections ${\text{CS}}_{k^{\prime }}$ for the estimation of survival with treatment. This choice allows to capture a greater number of treated patients, without unnecessary computational intensity. All estimation methods assume linearity and proportional hazards for $Z_{k}$ ’s effect on the outcome, leading to some model misspecification as the proportional hazards assumption does not hold for the marginal never-treat survival^16^ and the linearity assumption does not hold for the effect of $Z_{k}$ on post-treatment survival.

#### 3.1.5. Performance measures

The performances of the three models are evaluated by assessing the calibration. More specifically, we compare the estimated $\hat{{\text{RMST}}^{0}}(S_{k}\;|\;{\bar{Z}}_{k})$ , $\hat{{\text{RMST}}^{1}}(S_{k}\;|\;{\bar{Z}}_{k},Z_{k}^{*})$ , and $\hat{d}(S_{k}\;|\;{\bar{Z}}_{k},Z_{k}^{*})$ to the true values ${\text{RMST}}^{0}(S_{k}\;|\;Z(t))$ , ${\text{RMST}}^{1}(S_{k}\;|\;Z_{k},Z_{k}^{*})$ , and $d(S_{k}\;|\;Z(t),Z_{k},Z_{k}^{*})$ (as defined at equations (9) and (10)). This means that the true values take the place of the “observed values” which are usually used in calibration assessment.^17,18^ All comparisons between estimated and true values of the estimands are done for each patient i , at each cross-section ${\text{CS}}_{k}$ where the patient is active, for each run of the simulation.

Calibration is presented both graphically, by means of calibration curves, and numerically, by computing the root mean squared bias, as an overall summary measure of calibration. Root mean squared bias, which we will abbreviate to bias, has been indicated as a good summary of overall calibration into one number.^17,18^ It is calculated by taking the squared differences between the smoothed calibration curve and the diagonal line of perfect calibration,^18^ for each patient at each cross-section for each simulation run, and then taking the square root of the average over these squared differences. The smoothing of the calibration curve serves to implicitly marginalize ${\text{RMST}}^{0}(S_{k}\;|\;Z(t))$ and $d(S_{k}\;|\;Z(t),Z_{k},$ $Z_{k}^{*})$ over all possible future trajectories (across cross-sections and simulation runs).

#### 3.1.6. Software

All analyses were conducted using the statistical software R (version 4.1.3)^19^ with the packages survival,^20^ tidyverse,^21^ and data.table.^22^ Our simulation code is available at https://github. com/survival-lumc/CondSurvivalBenefit.

### 3.2. Simulation results

**Figure 2.**
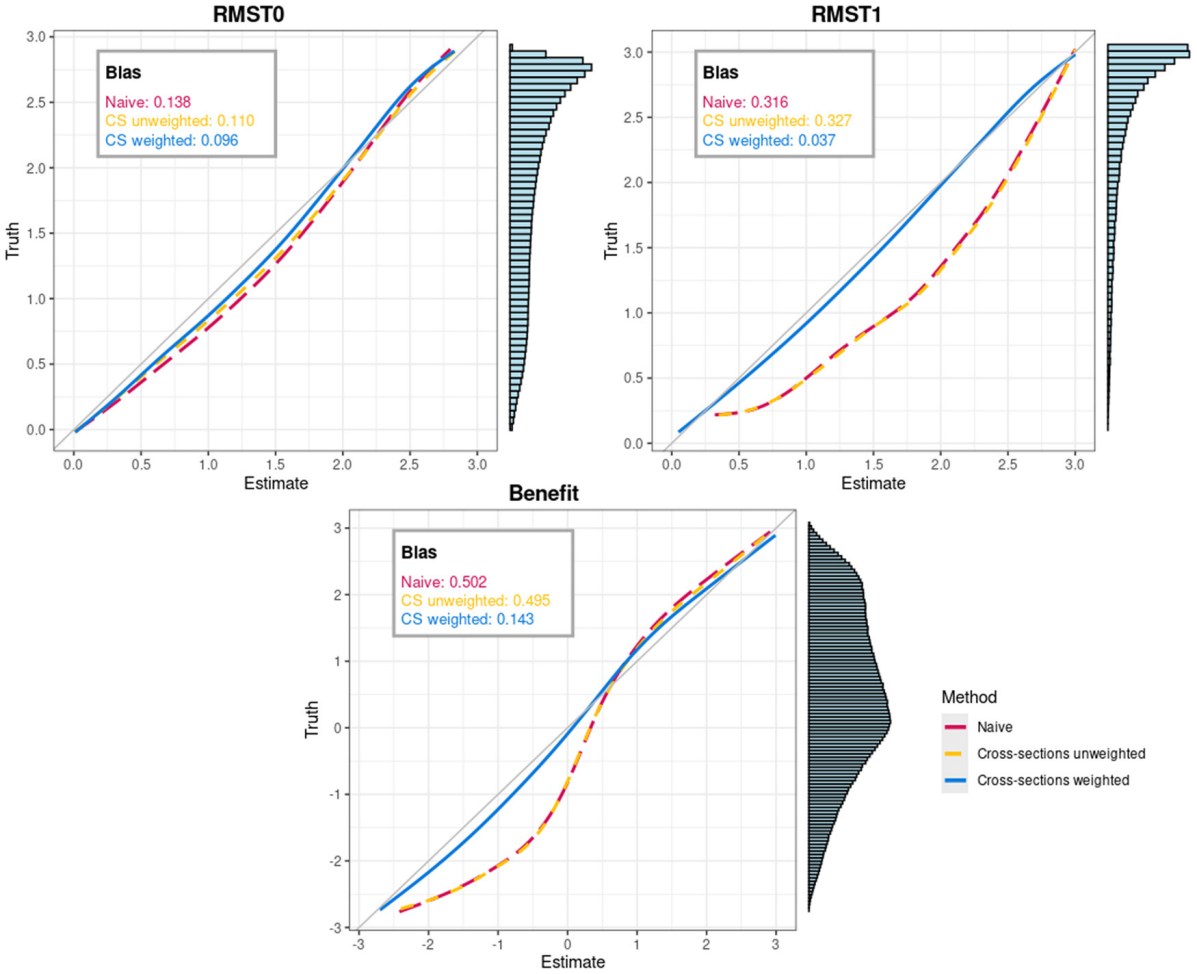
Calibration curves for the three estimation methods and root mean squared bias, here referred to as bias. In the calibration curve, the estimated and true value of the estimands ( ${\text{RMST}}^{0}$ , ${\text{RMST}}^{1}$ , and benefit) are compared for each patient, at each cross-section, for each simulation run. The smoothed estimate of the true RMST (or benefit) on the y -axis is plotted against the estimated RMST (or benefit) on the x -axis (averaged over simulation runs). Bias is calculated by taking the squared differences between the smoothed calibration curve and the diagonal line of perfect calibration, for each patient at each cross-section for each simulation run, and then taking the square root of the average over these squared differences. The three estimation methods are (i) the *naive* method, where ${\text{RMST}}^{0}$ and ${\text{RMST}}^{1}$ are estimated via Cox models that only take baseline measurements into account, without any adjustment (with baseline being, respectively, beginning of follow-up and time of treatment); (ii) the *unweighted cross-sections* method, which makes use of cross-sections but without the use of time-dependent weights; and (iii) the *weighted cross-sections* method, which is the sequential MSM approach we propose. RMST: restricted mean survival time; MSMs: marginal structural models.

We run the simulation 200 times, each time with a population of 2500 patients. Figure 2 presents the calibration plots and the resulting biases.

Overall, the proposed method yields lower bias than the two other methods. For the estimation of the restricted mean survival time without treatment, the proposed method outperforms the two other methods, as shown by both the root mean squared bias and the calibration plot. In particular, the proposed method appears to be able to better capture the RMST of those patients that are the sickest (i.e. those with lower true RMST ) and, therefore, more likely to receive treatment. This is expected, as the *naive* and *cross-section unweighted* methods ignore the time-varying nature of the confounder Z(t) beyond their respective baselines (time t=0 and time $t=CS_{k}$ ). As shown in the side histogram of the calibration plot, these very sick patients are less represented on the waiting list population we generated. Consequently, while the overall bias is lowest for the weighted method, the reduction in overall bias remains relatively small. The bias of the proposed model is not zero, which is also to be expected given that the marginal model that is used is misspecified with respect to the proportional hazards assumption.

In the estimation of the restricted mean survival time with treatment, our proposed method remains closer to the diagonal line of perfect calibration while the other methods deviate from it. This behavior is expected, as data were generated with a non-linear effect of the predictor on the log-hazards, while all three methods assume a linear effect. In the presence of some model misspecification, using weights yields estimates that better represent the full target population. The estimates of the *naive* and the *cross-section unweighted* methods, which do not reweight the population, are mainly based on patients with higher $Z_{k}$ , who are more likely to be treated. Since the effect of $Z_{k}$ is smaller in this group, the overall effect of $Z_{k}$ is underestimated. The *cross-section unweighted* and *naive* methods perform similarly, as expected. The *naive* method estimates post-treatment survival from treatment onward based on all treated patients. With a fine grid of cross-sections, the *cross-section unweighted* approach does the same, except for missing patients treated at calendar times not included in the grid. Consequently, the cross-sections, which are useful for the construction of the weights in the *cross-section weighted* approach, offer no advantage here.

The results for estimation of benefit follows its two components, with the proposed method outperforming the two naive approaches.

## 4. Application to allocation of liver transplants

We apply the proposed method to estimate the benefit of transplantation for patients with End-Stage Liver Disease in the Eurotransplant region. We use individual data on patients with chronic liver cirrhosis from the Eurotransplant registry, with patients being followed between 1 January 2007 and 31 December 2019. For our analysis, we use a subset of 9868 patients. A full description of the selection criteria is presented in Section C.1 in the Supplemental Material. Of these patients, 4998 were eventually transplanted, 3013 died before receiving a transplant and 1361 died within 3 years of being transplanted.

In this study, the goal is to build a model that, at any calendar date that a donor liver becomes available, can inform about the contrast between (i) survival probability over the next 3 years if the patient does not receive a transplant within 3 years, based on their individual current health status and (ii) survival probability over the next three years if the patient does get transplanted at that specific date, based on their individual current health and the quality of the donor liver being available for transplantation. The contrast is expressed as the difference of the RMSTs given by (ii) and (i), with a time horizon of 3 years. This contrast, namely the 3 years survival benefit, provides information about the potential gain in life-years (out of 3 life-years) obtained by allocating the available liver to this specific patient.

In the analysis, we make use of both recipient candidate and donor data. Data on the recipients consist of longitudinal measurements of serum bilirubin and creatinine, the international normalized ratio (INR), dialysis status (yes/no), and eligibility status as well as baseline information on age, blood group, gender, registration country, weight, and height. The longitudinal measurements are available from first eligibility to delisting, which could be due to death, transplantation, removal due to worsening of health conditions, removal due to improvement, cut-off date of the data extraction or loss to follow-up. The donor data consists of information on the donor livers: age of the donor at time of death, country of the donor, rescue liver offer (yes/no), donation after cardiac death (yes/no), and cause of death. All continuous variables were centered and standardized for the analysis (column “Overall” of Table 1 in Section C in the Supplemental Material presents their mean and standard deviation).

With slight abuse of terminology, we consider removal due to worsening of health conditions as death, as patients who are removed due to worsening will typically no longer be able to have access to a transplant as a source of treatment and their health will quickly deteriorate to death. Removal due to improvement, cut-off date of the data extraction or loss to follow-up are considered as a censoring mechanism that is independent conditional on baseline covariates, which here means “at cross-section” (see Section C.2 in the Supplemental Material for details). All times were rounded to the nearest 0.01 years.

Survival without treatment is estimated from K=120 evenly spaced calendar cross-section dates while survival with treatment is estimated from K=1200 evenly spaced calendar cross-section dates. Both sets of dates range from 1 January 2007 to 31 December 2018. Survival with and without liver transplant is estimated from these cross-sections onward. We use a finer set of dates for estimating survival with treatment to capture a greater number of treated patients. In contrast, a smaller set of dates is used to estimate of survival without treatment to avoid unnecessary computational intensity. This smaller set of dates is sufficient for the to estimation of survival without treatment, as there are many untreated patients at each cross-section. Patients are included in a cross-section if they are on the waiting list and are eligible for transplant right before that date, and have complete measurements of the covariates on that date (129 patients had some missing or invalid values across the longitudinal covariates).

In order to estimate the stabilized IPTW, as specified in (6), we fit two Cox models for the weights of the untreated patients and a complementary log-log regression for the weights of the treated patients, as described in equation (7). In the two Cox models, we include the time-fixed covariates age, sex, blood group, weight, and height and the time-varying covariates MELD score and dialysis twice within the last week (yes/no). The baseline hazards of the two models are stratified by recipient’s country of listing. In the complementary log-log regression, we include the same patient covariates as the Cox models, as well as treatment specific covariates: donor age, rescue liver offer (yes/no), cause of death, donation after cardiac death (yes/no), and proximity between transplantation center and donor liver (in three categories, i.e. same center, same country, or abroad). Cross-sections with multiple treatments were treated as separate cross-sections, that is, different copies of the patients were made, one for every treatment available at that time. We pool together the cross-sections and estimate a single common intercept, under the assumption that the composition of the cross-sections is similar through time. Weights are capped to the 99.99 percentile to exclude extreme values. The coefficients of the models can the found in Tables 2 to 4 in Section C.3 in the Supplemental Material. To check whether the positivity assumption is met, we looked into the distribution of the variables used in the weights estimation, stratified by treatment status over time, to assess common support between treated and untreated. Further details can be found in Section C.4 in the Supplemental Material.

We fitted our MSM as described in equation (5). For patients with transplant status equal to 0 (i.e. not yet transplanted), we include as patient-specific covariates age at time of cross-section, sex, weight, height, blood group, latest serum bilirubin measurement, latest serum creatinine measurement, latest INR measurement, dialysis twice within the last week (yes/no), and time spent on the waiting list. For patients with transplant status equal to 1 (i.e. transplanted), we include as patient-specific covariates age at time of cross-section, sex, latest MELD score, and dialysis twice within the last week (yes/no). Donor age, rescue liver offer (yes/no), cause of death, donation after cardiac death (yes/no), and proximity between transplantation center and donor liver (same three categories, i.e. same center, same country, or abroad) are considered as treatment-specific covariates. We assume no interactions between variables and we pool all K baseline hazards into one (in other words, we assume a common baseline hazard). We stratify the baseline hazard by recipient’s country of listing. The summary of the model is presented in Table 5 in Section C.5 in the Supplemental Material.

The estimated MSM is then applied to the same data, in order to estimate the three-years survival benefit on the waiting list patients. Estimations are made at the same cross-section dates, based on the patients’ covariates at those dates. For the donor characteristics, we sample with replacement from the donor data a set of K donors and match each donor to one cross-section.

**Figure 3.**
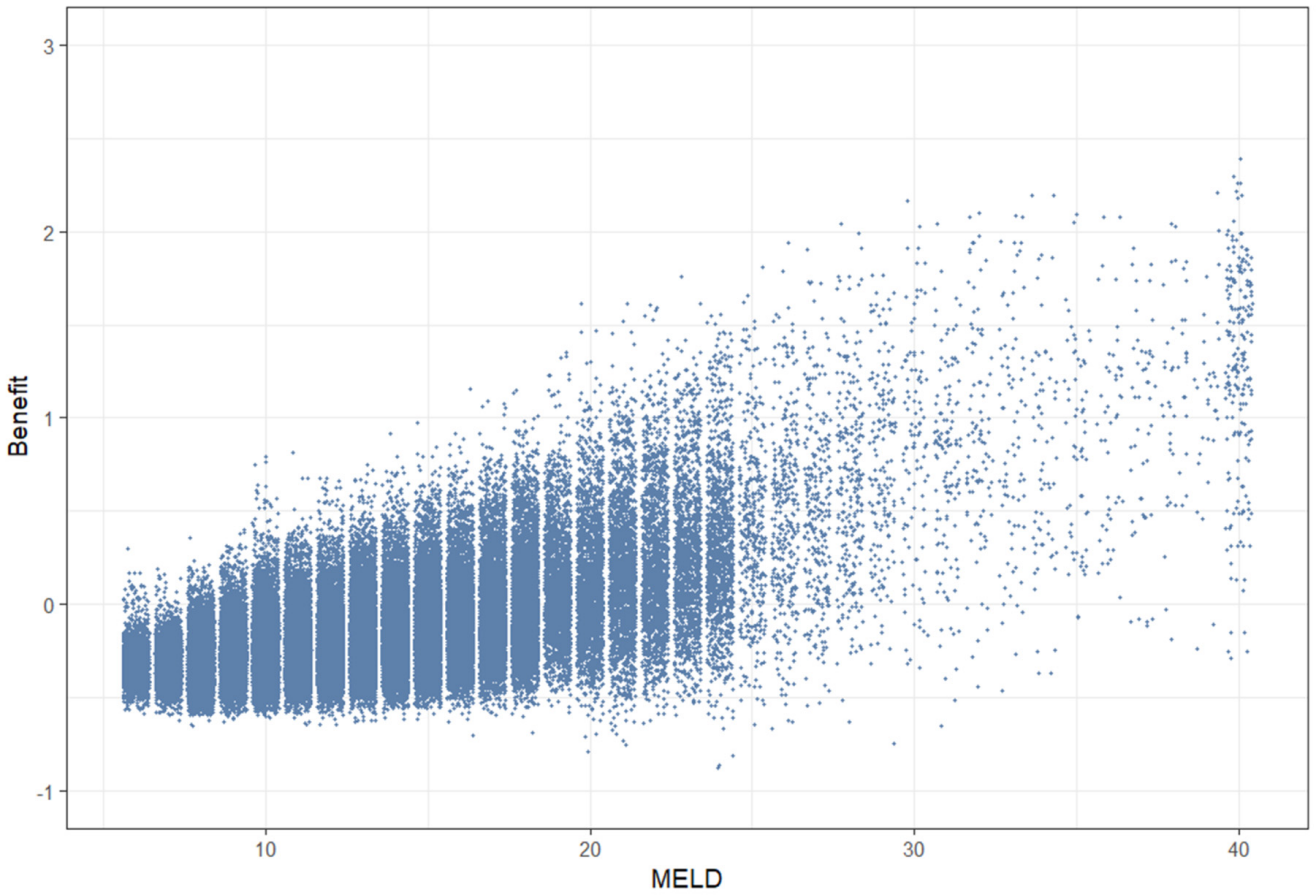
The Model for End-stage Liver Disease (MELD) score of each patient at each cross-section date is plotted against the respective estimated three-years survival benefit of that patient at that cross-section date. This means that one dot represents one cross-section-patient combination. To improve readability, random uniform noise ( ±0.4 ) was added to each integer MELD value. In this figure, it is possible to see how a MELD-based and a benefit-based prioritization rule would differ.

In Figure 3, the MELD score of each patient at each time they are included in one of the 120 cross-section used to estimate survival without treatment is plotted against the respective estimated three-years survival benefit of that patient at that cross-section date. This figure clearly highlights the differences between the two prioritization rules. The current prioritization rules in the Eurotransplant region rely on the MELD score which was created to estimate 3 months survival while on the waiting-list (hence before treatment), while our proposed method estimates the life-years gained with transplantation in the next 3 years. A MELD-based prioritization favors patients with the highest MELD scores (those on the far right of Figure 3), while a prioritization based on benefit, as estimated by our MSM, would favor patients with the highest estimated survival benefit (those at the top of Figure 3). While on average an increase in MELD corresponds to an increase in benefit, it is also true that if patients were to be allocated to transplant according to the MELD score (as is currently implemented in the Eurotransplant region), we would not be necessarily selecting the patient with most benefit from that liver: there are patients with MELD score between 20 and 30 who seem to have larger benefit than patients with MELD score between 30 and 40 and, vice versa, some patients with high MELD score do not seem to have a great survival benefit.

By selecting at each cross-section one patient who would be the top pick according to a benefit based allocation and one patient who would be the top pick according to a MELD-based allocation, we computed that, on average, 0.46 life-years (out of the next 3 life-years) could have been saved extra by using a benefit-based allocation system. Given that over the last 10 years almost 5000 patients in our dataset received a transplant, this means that up to 2300 life-years could have potentially been saved for these individuals.

## 5. Discussion

In this work, we propose a sequential MSM approach for the prediction of conditional survival benefit for patients on a waiting list at the moment a scarce intervention becomes available. To showcase the value of our method, we apply it to estimate the (potential) conditional survival benefit of liver transplantation for patients with End-Stage Liver Disease via individual patient data from the Eurotransplant registry. The simulation study shows that the proposed method outperforms simpler methods which either do not account for confounding or ignore the need to combine two different time scales.

The proposed method borrows the idea of making sequential cross-sections on the calendar time scale from previous work,^5,11^ where cross-sections were combined together with inverse probability of censoring weighting to estimate survival benefit for the treated population. In our work, however, cross-sections are used to obtain predictions of survival benefit that are applicable to all patients awaiting treatment, and not just to the treated population as by Gong and Schaubel,^5^ Schaubel et al.,^8,9^ and Gong and Schaubel^11^ These predictions can be used to rank patients awaiting for treatment based on benefit and may support allocation choices. It is important to underline that the use of cross-sections is motivated by the question at hand, namely: *which patients have the largest gain in life years if they receive treatment at this calendar date?* This question, where the calendar time at which treatment is given is key, is answered by estimating the average treatment effect for each patient conditional on both characteristics of the patient and characteristics of the treatment that came available at that specific date. While previous work has estimated the effect of transplantation for wait-listed patients,^23,24^ the treatment effect was estimated using only one time scale, hence targeting a different question, namely: *what is the average treatment effect for a wait-listed patient, conditional on some clinically relevant characteristics?* The two questions are not equivalent, as the population composition of the cohort at time of first eligibility list is different from the composition at given calendar dates. Our proposed method combines the two different time scales that are at place, thus answering our question of interest.

Compared to previous approaches, our method offers several advantages. First of all, survival with and without treatment can either be estimated separately (by means of separate baseline hazards, as demonstrated in the data application) or in one single model (by means of one single baseline hazard and including treatment as a time-dependent variable in the MSM), granting flexibility. Arguments pro and against both choices can be made. On the one hand, if treatment is estimated non-parametrically, fewer assumptions are needed in the model regarding its effect. On the other hand, one might argue that the proposed method estimates benefit as a difference of two estimands and not directly as a self-standing quantity.^25^ Flexibility in our method is also partially ensured by the use of inverse probability of treatment weighting, which allows for the separate handling of confounding and analysis in two different steps.^3^ Moreover, the cross-sections, which are used to target the correct population of interest, allow for the specification of interactions between treatment and time-varying covariates, which simpler MSMs cannot achieve.^26^

The cross-sections also ensure efficient use of longitudinal data.^6^ In particular, this use of the longitudinal data at hand, which considers measurements taken at multiple calendar dates as baselines, leads to a larger sample size compared to the one provided by one single time origin. This is also useful in light of estimation of weights, which are known to lead to greater variance. Previous work showed that the use of sequential trials leads to less extreme weights compared to simpler MSMs with only one time origin.^27^

In the real data application, we applied the estimated MSM to the same data at the same calendar dates, while doctors would want to use the estimated MSM to make predictions for future patients on the waiting list at different calendar dates. To do so, decisions must be made regarding the baseline hazards of the two treatment strategies. If the baseline hazards are estimated non-parametrically for each cross-section separately, they cannot be directly extrapolated to predictions at later dates, and assumptions are required. For example, one could assume that the latest baseline hazard best reflects recent mortality rates (for both treated and untreated patients) and use that for prediction. Alternatively, baseline hazards could be pooled across several cross-sections, perhaps assuming that cross-sections of the same calendar year share one common baseline hazard, and the latest pooled hazard could be used for future predictions. Another approach might involve modeling the calendar dates parametrically, rather than using them as strata, to capture mortality trends over time, allowing for prediction under the assumption of continuation of the identified trend. In our application, we used one common baseline hazard pooled across all cross-sections.

Our method also has some limitations, which should be acknowledged. Firstly, it is computationally intensive. The use of K cross-sections alone means combining K datasets together. The addition of time-dependent weights means further amplification of the data volume by a multiplicative factor. There is no clear indication of what is the best or most appropriate number of cross-sections, although some guiding principles have been provided to ensure a good combination of gain in information without making the model needlessly intensive from a computational perspective.^5^ Secondly, our method relies on fundamental assumptions commonly associated with causal inference approaches, including consistency, positivity, and conditional exchangeability as well as a correct specification of both the time-to-treatment model and the outcome model.^3^ While we have introduced a certain level of model misspecification in our simulation, by generating the time-dependent covariate Z(t) and the hazard $h^{0}(t)$ in such a way that the proportional hazard assumption is not guaranteed when using our proposed method, we have not extensively explored scenarios where the fitted model deviates in a more pronounced way from the true model (e.g. the scenario where some residual confounding remained unaddressed). Finally, our approach allows to maximize survival benefit for the population which is eligible at each specific calendar date when new treatments become available, by identifying those with the highest predicted benefit. However, a model designed to maximize survival benefit for the population across all calendar dates would likely be of greater interest to policymakers. Although this was not the focus of our current work, we believe our methods could be extended for this purpose if combined with a model predicting future treatment availability. Such a model would enable us to identify not only the patients with the highest current benefit but also those whose expected benefit would diminish or increase if they were to wait longer for treatments. This could be a promising venue for future research.

Despite being based on a relatively small cohort of 231 patients from four medical centers within the United States between 1991 and 1995,^28^ the MELD score continues to be utilized in the Eurotransplant area to prioritize patients for allocation of liver organs. In recent years, efforts have been made to update and improve the MELD score to better capture the characteristics of the Eurotransplant patients^29,30^ and, therefore, improving the prioritization rule for the Eurotransplant area. These improvements involve re-fitting the models and subsequently adjusting the coefficients of each MELD component. Our work further contributes to the aim of exploring and improving prioritization rules for the Eurotransplant area, by proposing a methodology to estimate survival benefit and therefore providing the tools needed to shift from a traditional “sickest first” allocation approach to a prioritization strategy that focuses on individuals with the highest potential benefit. Such an allocation system has already been in place in the UK since 2018, after the National Health Service gave a presentation over the potential of a benefit-based allocation,^31^ which was studied by means of a simulated allocation model. It is important to note that while our method has the potential to guide allocation choices, the current analysis was conducted on a specific subset of patients within the registry data. For actual implementation purposes, it is essential to include all wait-listed patients. Some variables with known high predictive value, such as sodium, albumin and oncological parameters, are currently not routinely collected in the Eurotransplant area. The addition of these variables to the prediction model would allow for more discriminative predictions. Further considerations will also be needed for particular subsets of patients, for example, patients seeking improved quality of life rather than life-saving treatment, who may need to be scored differently (i.e. not based of survival benefit). A simulated allocation model similar to LSAM (Liver Simulated Allocation Model), which was developed by the Scientific Registry of Transplant Recipients^32^ for the United Network for Organ Sharing region, is needed to more accurately evaluate how the waiting list composition would change by a change to benefit-based allocation and to get a more precise estimate of how many life-years could be saved with benefit-based allocation.

In conclusion, we propose the use of a sequential MSM approach for predicting conditional survival benefit for patients on a waiting list. By applying it to estimate the potential survival benefit of liver transplantation for patients with End-Stage Liver Disease, we demonstrate its value. Our method provides flexibility in the modeling of treatment effect and incorporates longitudinal data effectively. It offers insights for prioritizing patients based on benefit thus supporting allocation strategies. Further research is needed to explore model misspecification scenarios and to gain more insight on how benefit-based allocation would change the composition of the liver transplant waiting list.

## Supplemental Material

sj-pdf-1-smm-10.1177_09622802261420699 - Supplemental material for Estimating conditional survival benefit for the allocation of scarce resources Supplemental material, sj-pdf-1-smm-10.1177_09622802261420699 for Estimating conditional survival benefit for the allocation of scarce resources by Ilaria Prosepe, Nan van Geloven, Hans de Ferrante, Andries E Braat and Hein Putter in Statistical Methods in Medical Research
