# Supplementary material for: Estimating conditional survival benefit for the allocation of scarce resources
Source: Stat Methods Med Res. 2026 Feb 17;35(4):812–26. doi: 10.1177/09622802261420699 (PMC13161485; doi:10.1177/09622802261420699)
Supplement: sj-pdf-1-smm-10.1177_09622802261420699 - Supplemental material for Estimating conditional survival benefit for the allocation of scarce resources [file sj-pdf-1-smm-10.1177_09622802261420699.pdf]

Supplemental material

A.1 Identification of the causal estimand

We prove that the survival distributions at Equation (1) of the main text are identifiable and can be expressed in terms of hazard functions and weights, with the hazard functions defined as

$$\lambda_{0,k}(u \mid \bar{\boldsymbol{Z}}_{\boldsymbol{k}}) = \lim_{\delta \to 0} \frac{1}{\delta} P\big[u \le D_k < u+\delta \mid \bar{\boldsymbol{Z}}_{\boldsymbol{k}}, \mathcal{E}_k = 1, D_k \ge u, \bar{A}_k(u^-) \equiv 0\big]$$
$$\lambda_{1,k,\ell}(u \mid \bar{\boldsymbol{Z}}_{\boldsymbol{k}}) = \lim_{\delta \to 0} \frac{1}{\delta} P\big[u \le D_k < u+\delta \mid \bar{\boldsymbol{Z}}_{\boldsymbol{k}}, \mathcal{E}_k = 1, D_k \ge u, A_{k,\ell}(0) = 1\big],$$

and weights corresponding to the inverse probability of having followed the treatment strategies $\underline{a}_k(0) \equiv 0$ and $\underline{a}_{k,\ell}(0) \equiv 1$ respectively. In simpler words, we prove that the survival distributions at Equation (1) can be estimated on a pseudo-population where subjects are artificially censored as soon as they deviate from the respective treatment strategies and reweighted by the inverse probability of keep on adhering to those strategies over time.

We divide the interval $[0, u]$ into $0 = u_0 < u_1 < \cdots < u_J = u$, such that $u_j - u_{j-1} = \delta$ for $j = \{1, 2, \ldots, J\}$. We assume that treatment initiation and events can only happen on the times u_j's. We also assume the following order of events: $A_k(u_{-1}) = 0 \to I(D_k = u_{-1}) = 0 \to \bar{\boldsymbol{Z}}_{\boldsymbol{k}}(u_0) \to A_k(u_0) \to I(D_k = u_0) \to \bar{\boldsymbol{Z}}_{\boldsymbol{k}}(u_1) \to \ldots \to A_k(u_j) \to I(D_k = u_j) \to \bar{\boldsymbol{Z}}_{\boldsymbol{k}}(u_{j+1}) \to \ldots$, where $u_0 = 0$ and we use the convention $A_k(u_{-1}) = 0$ and $I(D_k = u_{-1}) = 0$ to indicate that the patient starts at each cross-section alive and untreated. We let $\mathscr{C}_k^0(u_j) = \{D_k^0 \ge u_{j+1}, \mathcal{E}_k = 1, \bar{\boldsymbol{Z}}_{\boldsymbol{k}}\}$, $\mathscr{C}_k(u_j, 0) = \{\bar{\boldsymbol{Z}}_{\boldsymbol{k}}, \mathcal{E}_k = 1, D_k \ge u_{j+1}, \bar{A}_k(u_j) \equiv 0\}$, $\mathscr{C}_k^{1,\ell}(u_j) = \{D_k^{1,\ell} \ge u_{j+1}, \mathcal{E}_k = 1, \bar{\boldsymbol{Z}}_{\boldsymbol{k}}\}$ and $\mathscr{C}_{k,\ell}(u_j, 1) = \{\bar{\boldsymbol{Z}}_{\boldsymbol{k}}, \mathcal{E}_k = 1, D_k \ge u_{j+1}, A_{k,\ell}(0) = 1\}$. We note that:

$$\begin{aligned}
P\{D_k^0 = u_j \mid \mathscr{C}_k^0(u_{j-1})\} &= \mathbf{E}_{\bar{\boldsymbol{Z}}_{\boldsymbol{k}}(u_j)} P\left\{D_k^0 = u_j \,\middle|\, \mathscr{C}_k^0(u_{j-1}), \bar{\boldsymbol{Z}}_{\boldsymbol{k}}(u_j)\right\} \\
&= \mathbf{E}_{\bar{\boldsymbol{Z}}_{\boldsymbol{k}}(u_j)} P\left\{D_k^0 = u_j \,\middle|\, \mathscr{C}_k(u_{j-1}, 0), \bar{\boldsymbol{Z}}_{\boldsymbol{k}}(u_j)\right\} \\
&= \mathbf{E}_{\bar{\boldsymbol{Z}}_{\boldsymbol{k}}(u_j)} \left[\mathbf{E}\left[\frac{I(\bar{A}_k(u_j) \equiv 0)}{P\{\bar{A}_k(u_j) \equiv 0 \mid \mathscr{C}_k(u_{j-1}, 0), \bar{\boldsymbol{Z}}_{\boldsymbol{k}}(u_j)\}} \,\middle|\, \mathscr{C}_k(u_{j-1}, 0), \bar{\boldsymbol{Z}}_{\boldsymbol{k}}(u_j) \right] \right. \\
&\qquad \left. \mathbf{E}\left[I(D_k^0 = u_j) \,\middle|\, \mathscr{C}_k(u_{j-1}, 0), \bar{\boldsymbol{Z}}_{\boldsymbol{k}}(u_j) \right] \right] \\
&= \mathbf{E}_{\bar{\boldsymbol{Z}}_{\boldsymbol{k}}(u_j)} \left[\mathbf{E}\left[\frac{I(\bar{A}_k(u_j) \equiv 0) \cdot I(D_k^0 = u_j)}{P\{\bar{A}_k(u_j) \equiv 0 \mid \mathscr{C}_k(u_{j-1}, 0), \bar{\boldsymbol{Z}}_{\boldsymbol{k}}(u_j)\}} \,\middle|\, \mathscr{C}_k(u_{j-1}, 0), \bar{\boldsymbol{Z}}_{\boldsymbol{k}}(u_j) \right] \right] \\
&= \mathbf{E}\left[\frac{I(\bar{A}_k(u_j) \equiv 0) \cdot I(D_k^0 = u_j)}{P\{\bar{A}_k(u_j) \equiv 0 \mid \mathscr{C}_k(u_{j-1}, 0), \bar{\boldsymbol{Z}}_{\boldsymbol{k}}(u_j)\}} \,\middle|\, \mathscr{C}_k(u_{j-1}, 0) \right] \\
&= \mathbf{E}\left[\frac{I(\bar{A}_k(u_j) \equiv 0) \cdot I(D_k = u_j)}{P\{\bar{A}_k(u_j) \equiv 0 \mid \mathscr{C}_k(u_{j-1}, 0), \bar{\boldsymbol{Z}}_{\boldsymbol{k}}(u_j)\}} \,\middle|\, \mathscr{C}_k(u_{j-1}, 0) \right]
\end{aligned}$$

where the second equality holds due to consistency and conditional exchangeability, the third equality holds due to positivity and to the fact that we have multiplied by a quantity that is equal to 1, the fourth equality holds due to conditional exchangeability, and the last equality holds due to consistency. Similarly:

$$\begin{aligned}
P\{D_k^{1,\ell} = u_j \mid \mathscr{C}_k^{1,\ell}(u_{j-1})\} &= P\left\{D_k^{1,\ell} = u_j \left|\mathscr{C}_k^{1,\ell}(u_{j-1}, 1)\right.\right\} \\
&= \mathbf{E}\left[\frac{I(\bar{A}_{k,\ell}(u_j) \equiv 1)}{P\{\bar{A}_{k,\ell}(u_j) \equiv 1 \,|\, \mathscr{C}_{k,\ell}(u_{j-1}, 1)\}}\middle| \mathscr{C}_{k,\ell}(u_{j-1}, 1)\right] P\left\{D_k^1 = u_j \left|\mathscr{C}_{k,\ell}(u_{j-1}, 1)\right.\right\} \\
&= \mathbf{E}\left[\frac{I(\bar{A}_{k,\ell}(u_j) \equiv 1) \cdot I(D_k^1 = u_j)}{P\{\bar{A}_{k,\ell}(u_j) \equiv 1 \,|\, \mathscr{C}_{k,\ell}(u_{j-1}, 1)\}}\middle| \mathscr{C}_{k,\ell}(u_{j-1}, 1)\right] \\
&= \mathbf{E}\left[\frac{I(\bar{A}_{k,\ell}(u_j) \equiv 1) \cdot I(D_k = u_j)}{P\{\bar{A}_{1,\ell}(u_j) \equiv 1 \,|\, \mathscr{C}_{k,\ell}(u_{j-1}, 1)\}}\middle| \mathscr{C}_{k,\ell}(u_{j-1}, 1)\right].
\end{aligned}$$

Keeping in mind that the probability of remaining treated once treatment is already imitated is, in this application, always equal to 1, it follows that

$$\begin{aligned}
S^0(u, S_k \mid \bar{\boldsymbol{Z}}_{\boldsymbol{k}}) &= \left(\prod_{j=1}^{J} 1 - P\left\{D_k = u_j \left|\mathscr{C}_k(u_{j-1}, 0)\right.\right\}\right)\left(\prod_{j=1}^{J} \frac{1}{P\left\{A_k(u_j) = 0 \left|\mathscr{C}_k(u_{j-1}, 0), \bar{\boldsymbol{Z}}_{\boldsymbol{k}}(u_j)\right.\right\}}\right) \\
S^{1,\ell}(u, S_k \mid \bar{\boldsymbol{Z}}_{\boldsymbol{k}}) &= \left(\prod_{j=1}^{J} 1 - P\left\{D_k = u_j \left|\mathscr{C}_{k,\ell}(u_{j-1}, 1)\right.\right\}\right) \frac{1}{P\left\{A_{k,\ell}(u_j) = 1 \left|\mathscr{C}_{k,\ell}(u_{-1}, 1), \bar{\boldsymbol{Z}}_{\boldsymbol{k}}\right.\right\}}
\end{aligned}$$

If we take the limit $\delta \to 0$, the first products on the right hand-side of the two equations become respectively the infinite products $\pi_{u' \leq u}\{1 - \mathrm{d}\Lambda_{0,k}(u' \mid \bar{\boldsymbol{Z}}_{\boldsymbol{k}})\}$ and $\pi_{u' \leq u}\{1 - \mathrm{d}\Lambda_{1,k,\ell}(u' \mid \bar{\boldsymbol{Z}}_{\boldsymbol{k}})\}$, where $\Lambda_k(u \mid \bar{\boldsymbol{Z}}_{\boldsymbol{k}}, a \cdot \boldsymbol{Z}^*, \bar{A}_k(u) \equiv a)$ is the integral from 0 to u of the hazard function

$$\begin{aligned}
\lambda_{0,k}(u \mid \bar{\boldsymbol{Z}}_{\boldsymbol{k}}) &= \lim_{\delta \to 0} \frac{1}{\delta} P\big[u \leq D_k < u + \delta \mid \bar{\boldsymbol{Z}}_{\boldsymbol{k}}, \mathcal{E}_k = 1, D_k \geq u, \bar{A}_k(u^-) \equiv 0\big] \\
\lambda_{1,k,\ell}(u \mid \bar{\boldsymbol{Z}}_{\boldsymbol{k}}) &= \lim_{\delta \to 0} \frac{1}{\delta} P\big[u \leq D_k < u + \delta \mid \bar{\boldsymbol{Z}}_{\boldsymbol{k}}, \mathcal{E}_k = 1, D_k \geq u, A_{k,\ell}(0) = 1\big].
\end{aligned}$$

A.2 Dealing with censoring

When the censoring mechanism cannot be assumed to be independent of D_k conditionally on $\boldsymbol{Z}_{\boldsymbol{k}}$, we can address dependent censoring in the same way as with treatment, by establishing identifiability conditions under which our estimand of interest remains identifiable despite the censoring mechanism. We can delineate assumptions for the case where: (i) while $\bar{A}(t) \equiv 0$, the patient's censoring chances at time t are influenced by $\bar{\boldsymbol{Z}}(t)$; (ii) when $A(t) = 1$, the patient's censoring chances at time t are influenced by $\bar{\boldsymbol{Z}}(T)$ and by the treatment type ℓ.

We let $C_k = C - S_k$. As we did in the previous section, we divide the interval $[0, u]$ into $0 = u_0 < u_1 < \cdots < u_J = u$, such that $u_j - u_{j-1} = \delta$ for $j = \{1, 2, \ldots, J\}$. We assume that treatment initiation, events and censoring can only happen on the times u_j. We assume the following ordering of events: $A_k(u_{-1}) = 0 \to I(C_k = u_{-1}) = 0 \to I(D_k = u_{-1}) = 0 \to \bar{\boldsymbol{Z}}_{\boldsymbol{k}}(u_0) \to A_k(u_0) \to I(C_k = u_0) \to I(D_k = u_0) \to \bar{\boldsymbol{Z}}_{\boldsymbol{k}}(u_1) \to \ldots \to A_k(u_j) \to I(C_k = u_j) \to I(D_k = u_j) \to \bar{\boldsymbol{Z}}_{\boldsymbol{k}}(u_{j+1}) \to \ldots$, where $u_0 = 0$ and we use the convention $A_k(u_{-1}) = 0$, $I(C_k = u_{-1}) = 0$ and $I(D_k = u_{-1}) = 0$ to indicate that the patient starts at each cross-section alive and untreated. Under this notation the identifiability conditions for $S^0(u, S_k \mid \bar{\boldsymbol{Z}}_{\boldsymbol{k}})$ are:

(i) **Consistency:**

- The counterfactual outcome D_k^0 is equal to the observed outcome D_k for those patients who die untreated; we write $D_k^0 = (D_k \mid \bar{A}(D_k) \equiv 0)$;
- The "what if the patients had stayed uncensored" outcome D is equal to the observed outcome X for those patients that die uncensored; we write $D_k = (X_k \mid C_k > D_k)$;

(ii) **Positivity:**

- The probability of being treated at time t given $\bar{\boldsymbol{Z}}(t)$, $\mathcal{E}_k = 1$, $A(t^-) \equiv 0$ and $D \geq t$ is less than 1 for all t;
- The probability of remaining uncensored at time t given $\bar{\boldsymbol{Z}}(t)$, $A(t^-) \equiv 0$ and $X \geq t$ is different from 0 for all t;

(iii) **Conditional exchangeability:**

- for every $u \geq 0$: $D_k^0 \perp\!\!\!\perp A_k(u) \mid \bar{\boldsymbol{Z}}_k(u), A_k(u^-) \equiv 0, D_k \geq u, \mathcal{E}_k = 1$;
- $I(D_k = u) \perp\!\!\!\perp I(C_k = u) \mid \bar{\boldsymbol{Z}}_{\boldsymbol{k}}(u), \mathcal{E}_k = 1, A_k(u) \equiv 0, X_k \geq u$

while the identifiability conditions for $S^1(u, S_k \mid \bar{\boldsymbol{Z}}_{\boldsymbol{k}})$ are

(i) **Consistency:**

- The counterfactual outcome $D_k^{1,\ell}$ is equal to the observed outcome D_k for those patients who are treated at cross-section k with treatment type ℓ; we write $D_k^{1,\ell} = (D_k \mid A_{k,\ell}(0) = 1)$;
- The "what if the patients had stayed uncensored" outcome D is equal to the observed outcome X for those patients that die uncensored; we write $D_k = (X_k \mid C_k > D_k)$;

(ii) **Positivity:**

- The probability of being treated at CS_k with treatment type ℓ given $\mathcal{E}_k = 1$ and $\bar{\boldsymbol{Z}}_k$ is greater than 0;

- The probability of remaining uncensored at time u given $\bar{\boldsymbol{Z}}_{\boldsymbol{k}}$, $A_{k,\ell}(0) = 1$ and $X_k \geq t$ is greater than 0 for all u

(iii) **Conditional exchangeability:**

- $D_k^{1,\ell} \perp\!\!\!\perp A_{k,\ell}(0) \,|\, \bar{\boldsymbol{Z}}_k, \mathcal{E}_k = 1$;
- $I(D_k = u) \perp\!\!\!\perp I(C_k = u) \,|\, \bar{\boldsymbol{Z}}_{\boldsymbol{k}}, \mathcal{E}_k = 1, A_{k,\ell}(0) = 1, X_k \geq u$

Note that, the first conditional exchangeability condition has changed from the one reported in the main text, conditioning on $X_k \geq u$ rather than $D_k \geq u$.

Under these conditions, the estimand at Equation (1) of the main text can be consistently estimated. This can be proved by arguing similarly to the previous section. Following the same steps as in the previous section, we can see that

$$
\begin{aligned}
P\{D_k^0 = u_j \mid \mathscr{C}_k^0(u_{j-1})\} &= \mathbf{E}_{\bar{\boldsymbol{Z}}_{\boldsymbol{k}}(u_j)} P\left\{D_k^0 = u_j \left| \mathscr{C}_k^0(u_{j-1}), \bar{\boldsymbol{Z}}_{\boldsymbol{k}}(u_j)\right.\right\} \\
&= \mathbf{E}_{\bar{\boldsymbol{Z}}_{\boldsymbol{k}}(u_j)} P\left\{D_k^0 = u_j \left| \mathscr{C}_k(u_{j-1}, 0), \bar{\boldsymbol{Z}}_{\boldsymbol{k}}(u_j)\right.\right\} \\
&= \mathbf{E}_{\bar{\boldsymbol{Z}}_{\boldsymbol{k}}(u_j)} \left[\mathbf{E}\left[\frac{I(\bar{A}_k(u_j) \equiv 0) \cdot I(D_k^0 = u_j)}{P\{\bar{A}_k(u_j) \equiv 0 \,|\, \mathscr{C}_k(u_{j-1}, 0), \bar{\boldsymbol{Z}}_{\boldsymbol{k}}(u_j)\}} \middle| \mathscr{C}_k(u_{j-1}, 0), \bar{\boldsymbol{Z}}_{\boldsymbol{k}}(u_j)\right]\right] \\
&= \mathbf{E}_{\bar{\boldsymbol{Z}}_{\boldsymbol{k}}(u_j)} \left[\mathbf{E}\left[\frac{I(\bar{A}_k(u_j) \equiv 0) \cdot I(D_k = u_j)}{P\{\bar{A}_k(u_j) \equiv 0 \,|\, \mathscr{C}_k(u_{j-1}, 0), \bar{\boldsymbol{Z}}_{\boldsymbol{k}}(u_j)\}} \middle| \mathscr{C}_k(u_{j-1}, 0), \bar{\boldsymbol{Z}}_{\boldsymbol{k}}(u_j)\right]\right] \\
&= \mathbf{E}_{\bar{\boldsymbol{Z}}_{\boldsymbol{k}}(u_j)} \left[\mathbf{E}\left[\frac{I(C_k > u_j) \cdot I(\bar{A}_k(u_j) \equiv 0) \cdot I(D_k = u_j)}{P\{C_k > u_j \,|\, \mathscr{C}_k(u_{j-1}, 0), \bar{\boldsymbol{Z}}_{\boldsymbol{k}}(u_j)\} P\{\bar{A}_k(u_j) \equiv 0 \,|\, \mathscr{C}_k(u_{j-1}, 0), \bar{\boldsymbol{Z}}_{\boldsymbol{k}}(u_j)\}} \middle| \mathscr{C}_k(u_{j-1}, 0), \bar{\boldsymbol{Z}}_{\boldsymbol{k}}(u_j)\right]\right] \\
&= \mathbf{E}_{\bar{\boldsymbol{Z}}_{\boldsymbol{k}}(u_j)} \left[\mathbf{E}\left[\frac{I(C_k > u_j) \cdot I(\bar{A}_k(u_j) \equiv 0) \cdot I(X_k = u_j, \Delta_k = 1)}{P\{C_k > u_j \,|\, \mathscr{C}_k(u_{j-1}, 0), \bar{\boldsymbol{Z}}_{\boldsymbol{k}}(u_j)\} P\{\bar{A}_k(u_j) \equiv 0 \,|\, \mathscr{C}_k(u_{j-1}, 0), \bar{\boldsymbol{Z}}_{\boldsymbol{k}}(u_j)\}} \middle| \mathscr{C}_k(u_{j-1}, 0), \bar{\boldsymbol{Z}}_{\boldsymbol{k}}(u_j)\right]\right] \\
&= \mathbf{E}\left[\mathbf{E}\left[\frac{I(C_k > u_j) \cdot I(\bar{A}_k(u_j) \equiv 0) \cdot I(X_k = u_j, \Delta_k = 1)}{P\{C_k > u_j \,|\, \mathscr{C}_k(u_{j-1}, 0), \bar{\boldsymbol{Z}}_{\boldsymbol{k}}(u_j)\} P\{\bar{A}_k(u_j) \equiv 0 \,|\, \mathscr{C}_k(u_{j-1}, 0), \bar{\boldsymbol{Z}}_{\boldsymbol{k}}(u_j)\}} \middle| \mathscr{C}_k(u_{j-1}, 0)\right]\right]
\end{aligned}
$$

where $\mathscr{C}_k(u, 0)$ and $\mathscr{C}_{k,\ell}(u, 1)$ are now defined respectively as $\{\bar{\boldsymbol{Z}}_{\boldsymbol{k}}, \mathcal{E}_k = 1, X_k > u, \bar{A}_k(u) \equiv 0\}$ and $\{\bar{\boldsymbol{Z}}_{\boldsymbol{k}}, \mathcal{E}_k = 1, X_k > u, \bar{A}_{k,\ell}(u) \equiv 1\}$ respectively (again $X_k = u$ replaced $D_k = u$). As for $P\{D_k^{1,\ell} = u_j \mid \mathscr{C}_k^{1,\ell}(u_{j-1})\}$, if we also modify the equalities in a similar way, we obtain:

$$
P\{D_k^1 = u_j \mid \mathscr{C}_k^{1,\ell}(u_{j-1})\} = \mathbf{E}\left[\frac{I(C_k > u_j) \cdot I(\bar{A}_{k,\ell}(u_j) \equiv 1) \cdot I(X_k = u_j, \Delta_k = 1)}{P\{C_k > u_j \,|\, \mathscr{C}_{k,\ell}(u_{j-1}, 0)\} \cdot P\{\bar{A}_{k,\ell}(u_j) \equiv 1 \,|\, \mathscr{C}_{k,\ell}(u_{j-1}, 1)\}} \middle| \mathscr{C}_{k,\ell}(u_{j-1}, 1)\right].
$$

Reasoning as in the previous section and taking the limit $\delta \to 0$, we obtain that the estimand at Equation (1) of the main text, can be estimated as the survival function given by the hazard function

$$\lambda_{0,k}(u \mid \bar{\boldsymbol{Z}}_{\boldsymbol{k}}) = \lim_{\delta \to 0} \frac{1}{\delta} P\big[u \leq X_k < u + \delta, \Delta_k = 1 \mid \bar{\boldsymbol{Z}}_{\boldsymbol{k}}, \mathcal{E}_k = 1, X_k \geq u, \bar{A}_k(u^-) \equiv 0\big]$$
$$\lambda_{1,k,\ell}(u \mid \bar{\boldsymbol{Z}}_{\boldsymbol{k}}) = \lim_{\delta \to 0} \frac{1}{\delta} P\big[u \leq X_k < u + \delta, \Delta_k = 1 \mid \bar{\boldsymbol{Z}}_{\boldsymbol{k}}, \mathcal{E}_k = 1, X_k \geq u, A_{k,\ell}(0) = 1\big]$$

on a pseudo-population where subjects have been reweighted by the inverse probability of having followed throughout the treatment strategy of interest until time horizon (see Equation (6) of the main text for the weights) and by the inverse probability of remaining uncensored until time horizon. Reweighting by the inverse probability of remaining uncensored can be carried out similarly to the reweighting by the inverse probability of having followed throughout treatment strategy $\underline{a}_k(0) \equiv 0$, by modeling the hazard of being censored instead of the hazard of being treated.

B Simulation study

Naive method We fit two Cox proportional hazards models. The first model is (with N indicating *naive* method)

$$\lambda_0^N(t \mid Z(0)) = \lambda_{0,0}^N(t) \exp\big(\beta_0^N Z(0)\big),$$

fitted on the data set that consists of the baseline measurements (at time $t = 0$) of the time-dependent covariate $Z(t)$, the survival time measured from baseline to death, censoring or treatment and the survival status that takes value one when the survival time is equal to death time. The second model is (with t' being time since treatment)

$$\lambda_1^N(t' \mid Z(T), Z^*) = \lambda_{0,1}^N(t') \exp\big(\beta_1^N Z(T) + \gamma^N Z^*\big),$$

fitted on the data that consists of treated patients only, their measurements of the time-dependent covariate at time of treatment T, the survival time measured from treatment to death or censoring and the survival status that takes value one when the survival time is equal to death time. We then use these quantities to estimate

$$\widehat{\text{RMST}}^0(S_k \mid \bar{Z}_k) := \int_0^L \exp\Big(-\int_0^t \hat{\lambda}_0^N(s \mid Z_k)\mathrm{d}s\Big)\mathrm{d}t$$
$$\widehat{\text{RMST}}^1(S_k \mid \overline{Z}_k, Z_k^*) := \int_0^L \exp\Big(-\int_0^{t'} \hat{\lambda}_1^N(s \mid Z_k, Z_k^*)\mathrm{d}s\Big)\mathrm{d}t'.$$

Cross-sections unweighted Similar to the proposed method, but without re-weighting. We fit two Cox proportional hazards models. The first model is (with CsU indicating the *cross-sections unweighted* method and u indicating time since cross-section)

$$\lambda_0^{CsU}(u \mid Z_k) = \lambda_{0,0}^{CsU}(u) \exp\big(\beta_0^{CsU} Z_k\big),$$

fitted on the data set that consists of, for all patients who are eligible at each cross-section CS_k and do not receive treatment at CS_k, their measurements of the time-dependent covariate $Z(t)$ at time of cross-sections CS_k (Z_k), the survival time measured from time of cross-section to death, censoring or treatment, and and the survival status that takes value one when the survival time is equal to death time (from cross-section). The second model is

$$\lambda_1^{CsU}(u \mid Z_k, Z^*) = \lambda_{0,1}^{CsU}(u) \exp\big(\beta_1^{CsU} Z_k + \gamma^{CsU} Z^*\big),$$

fitted on the data set that consists of, for all patients who are eligible at each cross-section CS_k and receive treatment at CS_k, their measurements of the time-dependent covariate $Z(t)$ at time of cross-sections CS_k (Z_k), the survival time measured from time of cross-section to death or censoring, and the survival status that takes value one when the survival time is equal to death time (from cross-section). We then use these quantities to estimate

$$\widehat{\text{RMST}}^0(S_k \mid \bar{Z}_k) := \int_0^L \exp\Big(- \int_0^u \hat{\lambda}_0^{CsU}(s \mid Z_k) \mathrm{d}s \Big) \mathrm{d}u$$
$$\widehat{\text{RMST}}^1(S_k \mid \overline{Z}_k, Z_k^*) := \int_0^L \exp\Big(- \int_0^u \hat{\lambda}_1^{CsU}(s \mid Z_k, Z_k^*) \mathrm{d}s \Big) \mathrm{d}u.$$

Note that, compared to Equation (4.5) in the main manuscript, we do not stratify per cross-section k but assume instead a common baseline hazard in all cross-sections.

Cross-sections weighted This is the proposed method. We fit the two Cox proportional hazards models $\lambda_0^{CsW}(u \mid Z_k)$ and $\lambda_1^{CsW}(u \mid Z_k, Z^*)$ (with CsW indicating the *cross-sections weighted* method) on the same data as for the *cross-sections unweighted*. However, each observation in the data is re-weighted, using weights specified in Equation (4.6) in the main manuscript.

C Real data analysis

C.1 Inclusion criteria

The MELD score is commonly employed to prioritize patients for liver allocation based on their short-term mortality risk. The predictive value of MELD was initially validated for individuals with chronic liver cirrhosis. Since candidates with different forms of end-stage liver disease may face under-prioritization by MELD score due to quality of life considerations or risks of irreversible disease progression, Eurotransplant (ET) has an exception point system in place to address these concerns. Actual prioritization is thus based on a combination of MELD score and exception points. In the data set this is reflected by the existence of two MELD score variables: “MELD” and “MELD (match)”. While “MELD” is simply derived by the values of its three components (creatinine, bilirubin and the international normalized ratio), the value of “MELD (match)” is originally computed as “MELD” but it may then be changed due to specific rules implemented by Eurotransplant. For example, Eurotransplant requires centers to re-certify MELD scores, with a frequency depending on the last reported value. In case MELD scores are not re-certified, the “MELD (match)” score is downgraded. Another example is provided by the exception points: if a patients receives exception points, the “MELD (match)” score is upgraded and this new score is subsequently used for allocation.

For our analysis we use a subset of the Eurotransplant registry data of the years 2007- 2019, which consists of patients with more homogeneous characteristics and clinically more similar to those for whom MELD score was originally intended. Cirrhotic patients aged $\geq$ 18 at first eligibility waiting for a deceased donor liver who did not receive a previous liver transplantation were included. Patients from countries whose allocation system is not MELD-based were excluded (MELD-based countries are Belgium, Germany and the Netherlands). Patients with exception points at listing or known to get exception points based on baseline characteristics were excluded. Patients with hepatocellular carcinoma (HCC) at listing or who developed HCC after listing were excluded (of these patients $> 90\%$ had it at listing). Patients who were concurrently listed for transplant of another organ (a heart, lung, pancreas or intestine) were also excluded. The final sample consisted of 9894 patients of which we then excluded 26 patients with missing data either in time-fixed covariates or with no information on the longitudinal covariates. A description of the final data set can be found at Table 1.

Table 1. Descriptives of the dataset. The means, standard deviations (SDs), missing data count and ranges reported in the table refer to the data in longitudinal format. In longitudinal data format, for each patient there is one row for each visit while on the waiting list and one row with the post-transplant values. The column *Overall* reports the means and standard deviations of the reported variables for all rows of the data set; the column *Before transplant* only considers the rows related to the visits while on the waiting list; the column *After transplant* only considers the rows with post-transplant values. Abbreviations: MELD = Model for End-stage Liver Disease, INR = international normalised ratio, DCD = donation after cardiac death.

Variable		Overall (9868 patients; 107,188 visits)	Before transplant (9868 patients; 102,190 visits)	After Transplant (4998 patients; 4998 visits)
**Recipient**				
Age	Mean (SD)	52.2 (9.6)	52.2 (9.6)	54.0 (9.2)
	Range	16.0, 77.0	16.0, 77.0	16.0, 77.0
Bloodgroup	A	43%	43%	44%
	AB	2.8%	2.5%	8.5%
	B	12%	12%	13%
	O	43%	43%	34%
Gender:	Female	36%	36%	30%
	Male	64%	64%	70%
Weight	Mean (SD)	78.6 (17.3)	78.4 (17.3)	81.8 (17.2)
	Range	31.0, 173.0	31.0, 173.0	36.0, 170.0
Height	Mean (SD)	172.2 (9.5)	172.2 (9.5)	173.7 (9.0)
	Range	110.0, 286.0	110.0, 286.0	130.0, 208.0
MELD (match)	Mean (SD)	16.9 (7.7)	16.6 (7.5)	23.5 (9.5)
	Range	6.0, 40.0	6.0, 40.0	6.0, 40.0
	(Missing)	27	27	0
MELD	Mean (SD)	17.7 (7.3)	17.4 (7.1)	23.7 (9.4)
	Range	6.0, 40.0	6.0, 40.0	6.0, 40.0
	(Missing)	128	128	0
Bilirubin (log)	Mean (SD)	1.4 (0.7)	1.4 (0.7)	1.9 (0.9)
	Range	0.0, 4.6	0.0, 4.6	0.2, 4.2
	(Missing)	131	131	0
INR (log)	Mean (SD)	0.9 (0.2)	0.9 (0.2)	1.0 (0.3)
	Range	0.4, 4.0	0.4, 4.0	0.6, 2.7
	(Missing)	135	135	0
Creatinine (log)	Mean (SD)	0.8 (0.4)	0.8 (0.4)	0.9 (0.4)
	Range	0.0, 2.9	0.0, 2.9	0.1, 2.9
	(Missing)	133	133	0
Dialysis	No	91%	91%	85%
	Yes	8.9%	8.6%	15%
Country	Belgium	9.6%	9.1%	18%
	Germany	86%	86%	73%
	Netherlands	4.8%	4.6%	8.4%
**Donor**				
Death cause	Other	3.8%		3.8%
	Anoxia	17%		17%
	CVA/stroke	60%		60%
	Trauma	19%		19%
Rescue	No	63%		63%
	Yes	37%		37%
Donor age	Mean (SD)	54.1 (16.6)		54.1 (16.6)
	Range	1.0, 98.0		1.0, 98.0
Graft DCD	No	90%		90%
	Yes	9.9%		9.9%
Destination	Abroad	15%		15%
	Same center	11%		11%
	Same country	75%		75%

C.2 Outcome

Eurotransplant collects data on transplant candidates from first eligibility to delisting (death, transplantation, removal due to worsening of health conditions, removal due to improvement or loss to follow-up) as well as post-transplant outcomes up to the time of death or loss to follow-up. In our analysis, while we can consider loss to follow-up and censoring due to cut-off date of the data extraction as independent censoring, which does not constitute an issue in our estimation procedures, the remaining events (removal due to worsening of heath conditions and removal due to improving) need more thought. Due to the illustrative nature of this real-data example, we decided to consider removal due to improvement (which occurred for 828 patients) as independent censoring and removal due to worsening as death. However, we recommend to address removal due to worsening and removal due to improvement as dependent censoring for the actual implementation of this method, as specified in Section A.2 of the Supplementary Materials.

C.3 Time-to-transplant models

Here we present the summaries of the two Cox time-to-transplant models used to estimate the inverse probability of treatment weights needed to estimate survival without treatment, as well as the logistic regression needed for the weights of the treated patients.

As specified at Equation (7), the numerator and denominator of the weights of the untreated patients are based on two Cox models. In the denominator model, time starts at first eligibility ($t = 0$) and uses time-dependent covariates, which means that the whole longitudinal history of each patient is taken into account. In Table 2, we present a summary of the model where the coefficients represent the effect of the respective covariates on time-to-treatment. For those variables that change over time, the coefficient represent the effect of the latest measurement. In the nominator model, for each given cross-section date, time is measured from there onwards and only the values of the covariates at that specific cross-section dates are included in the model. Table 3 presents a summary of the numerator model where the coefficients represent the effect of the value of the respective covariates on time-to-treatment.

The denominator of the weights of the treated patients is based on a logistic model with a `cloglog` link. In Table 4, we present a summary of the model where the coefficients represent the effect of the covariates' measurements at each cross-section on treatment chances.

Note that, in all models, continuous variables have been centered and scaled. This means that the coefficients presented in the tables represent effects belonging to an increase of one standard deviation of the covariates, rather than one unit. By dividing the coefficient in the table by the standard deviation of a particular covariate, it is possible to estimate the effect of the variable per unit. The value of the standard deviation may be found in Table 1, in the column "Overall".

Table 2. Summary of the Cox model for the denominator of the inverse probability of treatment weights for the untreated strategy. Continuous variables have been centered and scaled. Abbreviations: SE = standard error, HR = hazard ratio, MELD = Model for End-stage Liver Disease.

	**Coefficient**	**SE**	**HR**	**P-value**
Age	0.122	0.015	1.130	<0.001
Weight	0.062	0.016	1.064	<0.001
Height	0.116	0.020	1.123	<0.001
MELD (match) (time-varying)	1.472	0.016	4.358	<0.001
Gender				
Female				
Male	0.029	0.041	1.030	0.476
Dialysis (time-varying)				
No				
Yes	-0.259	0.046	0.772	<0.001
Blood group				
A				
AB	1.242	0.055	3.461	<0.001
B	0.093	0.045	1.098	0.037
O	-0.406	0.033	0.666	<0.001

Table 3. Summary of the Cox model for the numerator of the inverse probability of treatment weights for the untreated patients. Continuous variables have been centered and scaled. Abbreviations: SE = standard error, HR = hazard ratio, MELD = Model for End-stage Liver Disease.

	**Coefficient**	**SE**	**HR**	**P-value**
Age	0.006	0.017	1.006	0.943
Weight	0.219	0.020	1.245	0.009
Height	-0.087	0.026	0.917	0.438
MELD (match)	1.411	0.026	4.100	<0.001
Gender				
Female				
Male	0.323	0.051	1.381	0.157
Dialysis				
No				
Yes	-1.039	0.105	0.354	0.001
Blood group				
A				
AB	1.569	0.084	4.801	<0.001
B	0.073	0.058	1.076	0.751
O	-0.353	0.039	0.702	0.053

Table 4. Summary of the cloglog model for the denominator of the inverse probability of treatment weights for the treated patients. Continuous variables have been centered and scaled. Abbreviations: SE = standard error, HR = hazard ratio, MELD = Model for End-stage Liver Disease.

	Coefficient	SE	HR	P-value
**Recipient**				
Waiting time	-0.995	0.039	0.370	<0.001
Age	0.160	0.016	1.173	<0.001
Weight	0.056	0.017	1.057	0.001
Height	0.092	0.021	1.097	<0.001
MELD (match)	1.672	0.016	5.323	<0.001
Gender				
Female				
Male	0.081	0.041	1.085	0.049
Dialysis				
No				
Yes	-0.400	0.047	0.670	<0.001
Blood group				
A				
AB	1.107	0.056	3.027	<0.001
B	0.110	0.045	1.116	0.015
O	-0.320	0.033	0.726	<0.001
**Liver quality**				
Donor age	0.027	0.018	1.028	0.132
Metabolic disease	0.028	0.079	1.029	0.722
Graft DCD				
No				
Yes	0.063	0.062	1.065	0.311
Death cause				
Other				
CVA stroke	-0.004	0.078	0.996	0.957
Trauma	-0.013	0.082	0.987	0.876
Anoxia	0.005	0.083	1.005	0.953
Rescue				
No				
Yes	0.579	0.036	1.785	<0.001
Destination				
Abroad				
Same country	-0.038	0.042	0.963	0.360
Same center	-0.019	0.069	0.981	0.780
**Interactions**				
MELD (match) : Donor age	-0.029	0.011	0.971	0.007
MELD (match) : Rescue	-1.006	0.029	0.366	<0.001
Age : Donor age	0.101	0.015	1.107	<0.001
Weight : Donor age	0.043	0.017	1.044	0.010
Height : Donor age	-0.029	0.017	0.971	0.092

C.4 Positivity

In the main text we delineated three identifiability conditions which are needed for the estimation of the quantity of interest. Among these conditions is positivity, asserting that, at any given time t: (i) the probability of receiving treatment conditional on patient's history up to time t is less than 1; the probability of receiving a specific treatment type conditional on patient's history up to time t is greater than 0. In this section we discuss the plausibility of this assumption in the context of the presented data application.

Firstly, let us recall the distinction between structural and practical positivity (1, Fine Point 13.1). Structural positivity means that both treatment choices are realistically feasible across subgroups of the population (the probability of being treated at time t is in the open interval $(0, 1)$ for all t, all subgroups and all treatment types). In the data application, structural positivity is plausible based on the following medical considerations. Patients on the waiting list are considered eligible for treatment, implying that, by definition, they have a chance > 0 to receive treatment. Their chance is never equal to 1, as livers are scarce and patients may be unlucky and not be assigned a donor before receiving treatment. Additionally, the availability of treatment is subject to variability and constraints: sometimes there is more availability of donor livers at a specific transplantation center (so patients with lower priority may receive treatment), sometimes they are particularly scarce (so even patients who would normally have high priority may die before they have access to treatment). Sometimes the first pick recipient candidate cannot reach the transplant facility soon enough and the liver is given to patients who have lower priority but are geographically closer.

Besides structural positivity, we also want to assess whether practical positivity holds in the data set at hand, i.e. that all treatment strategies are actually observed across subgroups in the data set. In Figure C.4.1, we give an overview of the distribution of the patient variables that are considered confounders of the relationship between treatment and outcome, stratified by treatment status at two different time points since first eligibility (which is the scale used for the weights). This examination should ideally be conducted at all (or a fine grid of) time points and for all possible treatment types. For brevity, we present the findings for these two points only and stratify for treatment yes/no only. This examination indicates no apparent issues with positivity for most variables. The only variable approaching zero density for certain values in either the treated or untreated group is MELD, which aligns with expectations. In Figure C.4.2, we zoom in on this variable to demonstrate that its density does not actually reach zero at these time points. We also extend the MELD examination to later time points. At later time points, the population itself has changed: most people with high MELD have either already been transplanted or have died. People who have a very high MELD and are on the waiting list at 1 year or at 2 years after listing are most likely patients who were healthier at baseline. Due to the lower sample size of patients with high

MELD at a later time point (in terms of time since baseline), we see that for some of the high MELD values the density function does indeed go to zero for the untreated group. This means that our model may potentially give less accurate estimates of untreated risks for patients with high MELD values at those later time points.

For fully non-parametric estimation, positivity would need to hold for much more granular defined subgroups, taking into account the multitude of variables, time points and donor characteristics. Defining such small subsets of patients entirely alike and estimating risks separately on these is not feasible in our limited size data set. For this reason, some modeling assumptions are needed. In our final MSM (Section C.5 of the Supplementary Materials) we assume no interactions between variables other than allowing for the effect of each patient variable to change after treatment. For the estimation of the time-to-treatment model, needed for the weights, we rely on the assumptions detailed in the main text and in Section C.3 of these Supplementary Materials. Under the proposed estimation method for the weights (Section C.3 of the Supplementary Materials), we only need to concern us with practical positivity being met for each single patient covariate separately. Likewise, we do not necessarily need to observe every single value of the MELD score for both the treated and untreated groups at each time point, as some level of extrapolation is permitted by the modeling choices made.

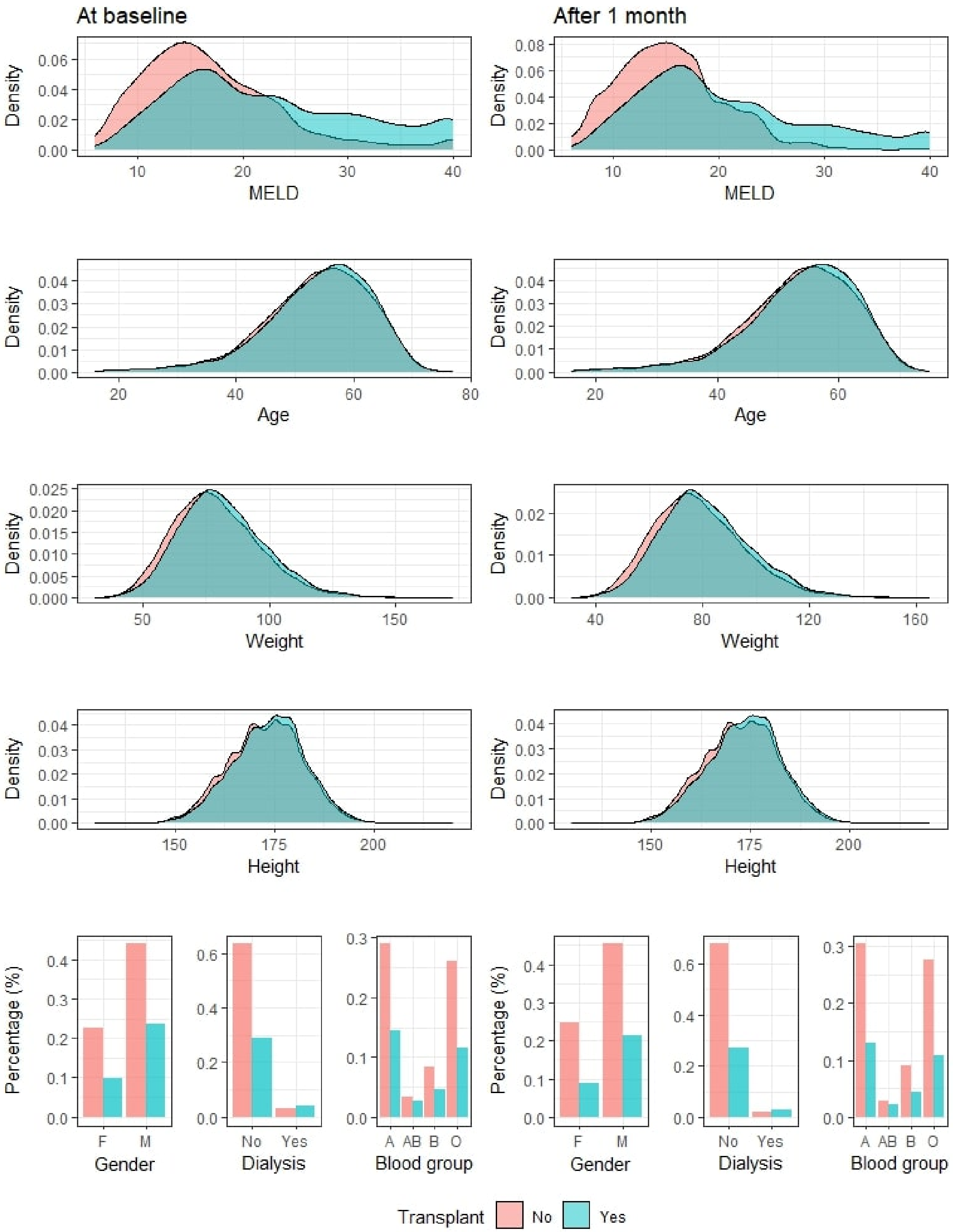

Figure C.4.1. Distribution of the variables included in the time-to-treatment model for the weights, stratified by treatment status at two different time points since first eligibility. Patients are considered treated if they receive treatment at the indicated time points (baseline or after 1 month), allowing for a grace period of one week. Patients that are still untreated at the end of the grace period are included in the untreated group.

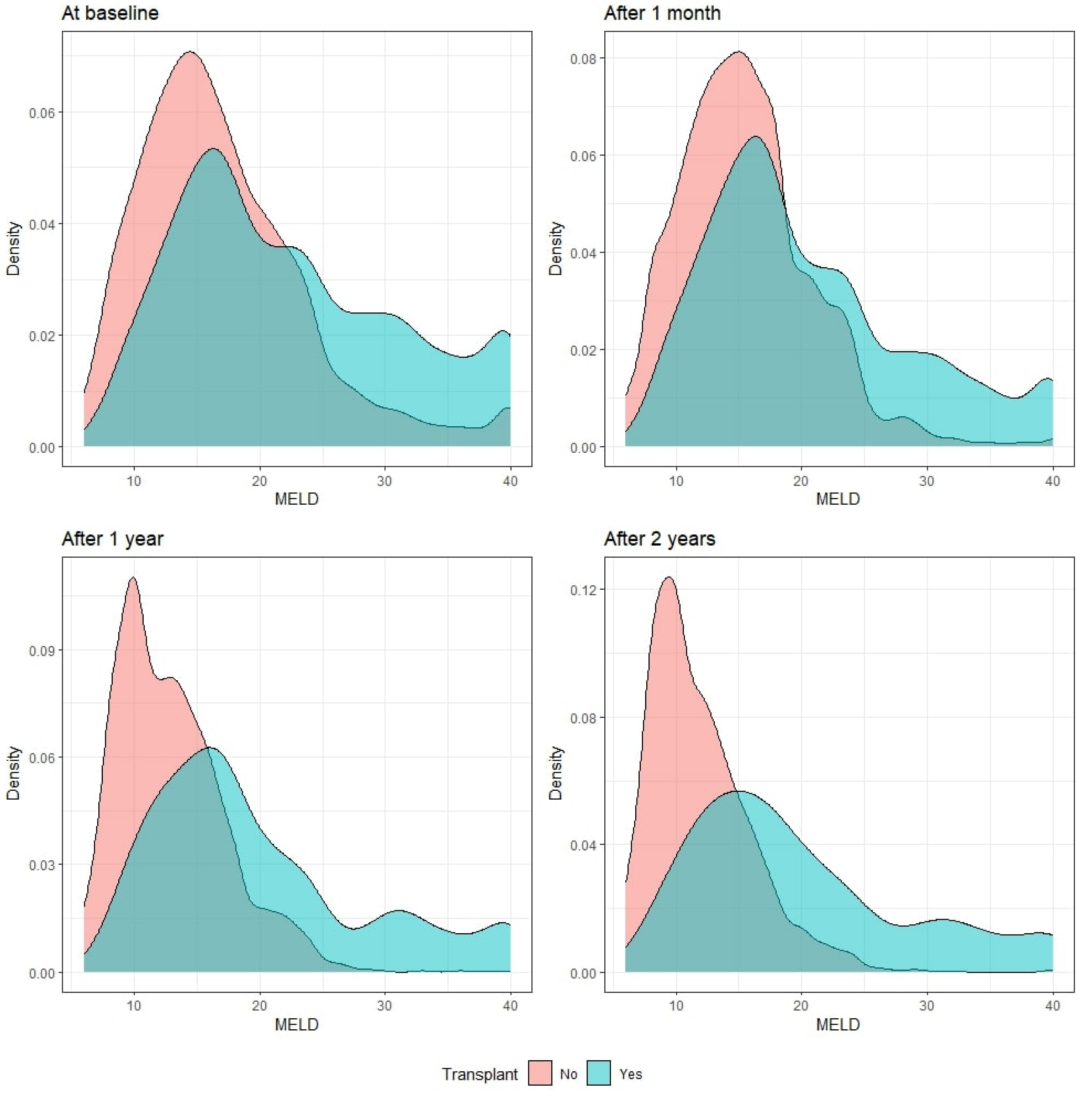

Figure C.4.2. Distribution of the MELD score variable, stratified by treatment status at four different time points since first eligibility. Patients are considered treated if they receive treatment at the indicated time points (baseline, after 1 month, after 1 year or after 2 years), allowing for a grace period of one week. Patients that are are still untreated at the end of the grace period are included in the untreated group.

C.5 Marginal structural model: time-to-event model

Table 5 presents the summary of our final survival time-to-event model, where the coefficients are estimated after the re-weighting. The coefficients reported below represent the effect of the covariates at a given calendar date on survival. Once again, continuous variables have been centered and scaled. To estimate the effect of each variable per unit, one needs to divide the coefficients in the table by the standard deviation (Table 1, column "Overall").

Table 5. Summary of the marginal structural model. Continuous variables have been centered and scaled. Abbreviations: SE = standard error, HR = hazard ratio, INR = international normalised ratio, WL = waiting list, PT = post transplant, DCD = donation after cardiac death, CVA = cerebrovascular accident.

	Coefficient	SE	HR	P-value
**Recipient (before transplant)**				
Age	0.366	0.008	1.442	<0.001
Weight	-0.025	0.009	0.976	0.044
Height	-0.080	0.010	0.923	<0.001
Bilirubin (log)	0.456	0.010	1.577	<0.001
INR (log)	0.360	0.011	1.433	<0.001
Creatinine (log)	0.324	0.014	1.382	<0.001
WL-time (log)	-0.162	0.013	0.851	<0.001
Gender				
Female				
Male	0.241	0.020	1.273	<0.001
Dialysis				
No				
Yes	0.124	0.059	1.132	0.250
Blood group				
A				
AB	-0.637	0.089	0.529	<0.001
B	0.337	0.022	1.400	<0.001
O	-0.042	0.015	0.959	0.032
**Recipient (after transplant)**				
Age-PT	0.083	0.027	1.087	0.002
MELD	0.180	0.028	1.197	<0.001
Gender				
Female				
Male	0.066	0.052	1.068	0.221
Dialysis				
No				
Yes	0.210	0.070	1.233	0.005
**Donor**				
Donor age	0.166	0.027	1.181	<0.001
Graft DCD				

No				
Yes	0.365	0.124	1.440	0.003
Death cause				
Other				
CVA stroke	-0.104	0.127	0.901	0.420
Trauma	-0.038	0.134	0.962	0.777
Anoxia	-0.169	0.138	0.844	0.228
Rescue				
No				
Yes	0.156	0.073	1.169	0.034
Destination				
Abroad				
Same country	0.010	0.072	1.010	0.894
Same center	-0.154	0.137	0.857	0.272

References

[1] Hernán M and Robins JM. *Causal Inference*. Chapman & Hall/CRC Monographs on Statistics & Applied Probability, Boca Raton: Chapman & Hall/CRC, 2021. ISBN 978-1-4200-7616-5.
